# Cold-induced vasodilation response in a Japanese cohort: insights from cold-water immersion and genome-wide association studies

**DOI:** 10.1186/s40101-023-00319-2

**Published:** 2023-03-08

**Authors:** Yoshiki Yasukochi, Toshihiro Sera, Taiki Kohno, Yusuke Nakashima, Musashi Uesugi, Susumu Kudo

**Affiliations:** 1 Department of Genome Analysis, Institute of Biomedical Science, Kansai Medical University, 2-5-1 Shin-Machi, Hirakata, Osaka 573-1010 Japan; 2 Department of Mechanical Engineering, Faculty of Engineering, Kyushu University, 744 Motooka, Nishi-Ku, Fukuoka, 819-0395 Japan; 3 Department of Mechanical Engineering, Graduate School of Engineering, Kyushu University, 744 Motooka, Nishi-ku, Fukuoka, 819-0395 Japan; 4 Graduate School of Systems Life Science, Kyushu University, 744 Motooka, Nishi-ku, Fukuoka, 819-0395 Japan

**Keywords:** Cold-induced vasodilation, Genome-wide association study, Japonica array, Single-nucleotide polymorphism, Wavelet analysis

## Abstract

**Background:**

Cold-induced vasodilation (CIVD) occurs after blood vessels in the skin are constricted due to local cold exposure. Although many CIVD studies have been conducted, the underlying molecular mechanisms are yet to be clarified. Therefore, we explored genetic variants associated with CIVD response using the largest-scale dataset reported to date in a CIVD study involving wavelet analysis; thus, the findings improve our understanding of the molecular mechanisms that regulate the CIVD response.

**Methods:**

We performed wavelet analysis of three skin blood flow signals [endothelial nitric oxide (eNO)-independent, eNO-dependent, and neurogenic activities] during finger cold-water immersion at 5 °C in 94 Japanese young adults. Additionally, we conducted genome-wide association studies of CIVD using saliva samples collected from the participants.

**Results:**

We found that the mean wavelet amplitudes of eNO-independent and neurogenic activities significantly increased and decreased prior to CIVD, respectively. Our results also implied that as many as ~ 10% of the Japanese subjects did not show an apparent CIVD response. Our genome-wide association studies of CIVD using ~ 4,040,000 imputed data found no apparent CIVD-related genetic variants; however, we identified 10 genetic variants, including 2 functional genes (*COL4A2* and *PRLR*) that are associated with notable blunted eNO-independent and neurogenic activity responses in individuals without CIVD response during local cold exposure.

**Conclusions:**

Our findings indicate that individuals without CIVD response differentiated by genotypes with *COL4A2* and *PRLR* genetic variants exhibited notable blunted eNO-independent and neurogenic activity responses during local cold exposure.

**Supplementary Information:**

The online version contains supplementary material available at 10.1186/s40101-023-00319-2.

## Background

Physiological thermoregulatory systems that act against extreme cold are essential for the survival of organisms. For example, constriction of skin vessels occurs in response to local cold exposure to minimize heat loss in the human body, resulting in attenuated skin blood flow (SkBF). After several minutes of cold exposure, peripheral blood flow increases due to subsequent vasodilation, known as “cold-induced vasodilation” (CIVD) or “hunting reaction” [1]. CIVD is thought to play an important role in biological defense against local cold-related injuries, such as frostbite of the fingers, although the underlying mechanisms of CIVD remain unclear [2]. Understanding these mechanisms could help prevent cold injuries. Interestingly, the CIVD response reportedly varies among ethnic groups; however, it is difficult to distinguish between the effects of genetic adaptation to cold environments (i.e., genetically fixed resistance to such environments in a population or ethnic group) and physiological acclimatization caused by cold stress (i.e., plastic physiological changes in an individual due to chronic or repeated cold stress) [2]. Although the timing of CIVD responses estimated using SkBF in the middle finger can be reproducible [3], the heterogeneity of CIVD responses among fingers and toes has been reported previously [4]. Such complexity increases the difficultly of elucidating the underlying mechanism of CIVD. In previous studies, wavelet analysis of SkBF measured using a laser Doppler velocimeter has been applied to examine CIVD responses because the wavelet transform can divide the measured perfusion signals into endothelial, neurogenic, myogenic, respiratory, and cardiac oscillatory components [5]. Such analysis allows researchers to estimate the activities of these components via a noninvasive monitoring technique.

Several hypotheses on the physiological mechanism of CIVD have been proposed. It has been suggested that CIVD is caused by neurogenic activity, such as the cessation of adrenergic neurotransmission, the release of dilating substances [e.g., nitric oxide (NO)] in the endothelium of blood vessels, and myogenic activity, such as the relaxation of the smooth muscle cells [2, 6–8]. Our previous study revealed that both neurogenic activity and endothelial NO (eNO)-independent activity may affect the finger SkBF response during immersion in cold water at 5 °C [9]. Indeed, it is possible that eNO-independent activity derived from endothelium-derived hyperpolarizing factor (EDHF) may affect the vasodilation of small vessels [10, 11]. Therefore, it is important to determine which molecules involved in eNO-independent and neurogenic activities primarily affect the CIVD response.

To elucidate the underlying physiological mechanism of CIVD, a genotype–phenotype association study is required because physiological functions are systematically regulated by interactions among various molecules. Although many CIVD studies have been conducted, the association between genetic variants and CIVD has yet to be revealed. This might be because substantial experimental effort is required to collect wavelet analysis data on SkBF; indeed, to the best of our knowledge, wavelet analysis of > 40 individuals has yet to be performed [12]. Here, we conducted such analysis using 94 study subjects. To quantify the magnitude of CIVD, wavelet analysis of SkBF signals was implemented to measure the magnitude of three possible vascular regulatory factors: eNO-independent, eNO-dependent, and neurogenic activities.

As mentioned, whether genetic polymorphism is attributable to the interethnic variability of the CIVD response is debatable [2]. To address this issue, we must explore the association between genetic polymorphisms and CIVD-related phenotypes to clarify the contribution of genetic variants to CIVD, which will provide crucial insights into the interethnic variability of the CIVD response. Therefore, we aimed to identify genetic variants related to CIVD and conducted genome-wide association studies (GWASs) on CIVD in 94 young Japanese adults. Our study is the first in which a finger cold-water immersion experiment and GWAS were performed, and we present the largest dataset in the field of CIVD to date. Moreover, our results begin to elucidate the underlying mechanism of CIVD by revealing the molecular functions related to the CIVD response.

## Methods

### Study subjects

In total, 94 healthy Japanese volunteers (93 males and 1 female; mean age ± standard error: 22.3 ± 0.1 years; height: 171.8 ± 0.6 cm; weight: 64.0 ± 1.1 kg) were recruited from Kyushu University (Fukuoka, Japan). The participants were nonsmokers and were not taking any medication. We refer to this cohort as the “JPQ cohort.” Since wavelet amplitudes of SkBF signals and anthropometric data such as body mass index and weight in the one female were not deviated from the corresponding measurements in males (Additional file 1: Table S1), we used the genotype and measurement data of the female for further analyses.

### Experimental cold-water immersion of fingers

The attenuated SkBF of the 94 study subjects was measured using laser Doppler flowmetry (FLO-C1, OMEGAWAVE). A probe for SkBF (ML type, OMEGAWAVE) and a thermistor for sensing temperature (TSD202F, BIOPAC Systems, Inc., USA) were attached to the ventral aspect of the distal phalanx of the middle finger at room temperature (25 °C). Before the experiment began, the subjects had rested in the sitting position for at least 30 min until all parameters to be examined were stable, and they further rested quietly for 10 min to collect baseline data. Subsequently, the middle finger of the participants was immersed up to the second phalanx for 30 min in a custom-made circulating cold water bath (5 °C). We used the 60-s moving average filter of the measurements for SkBF and finger skin temperature to avoid small fluctuations.

**Fig. 1:**
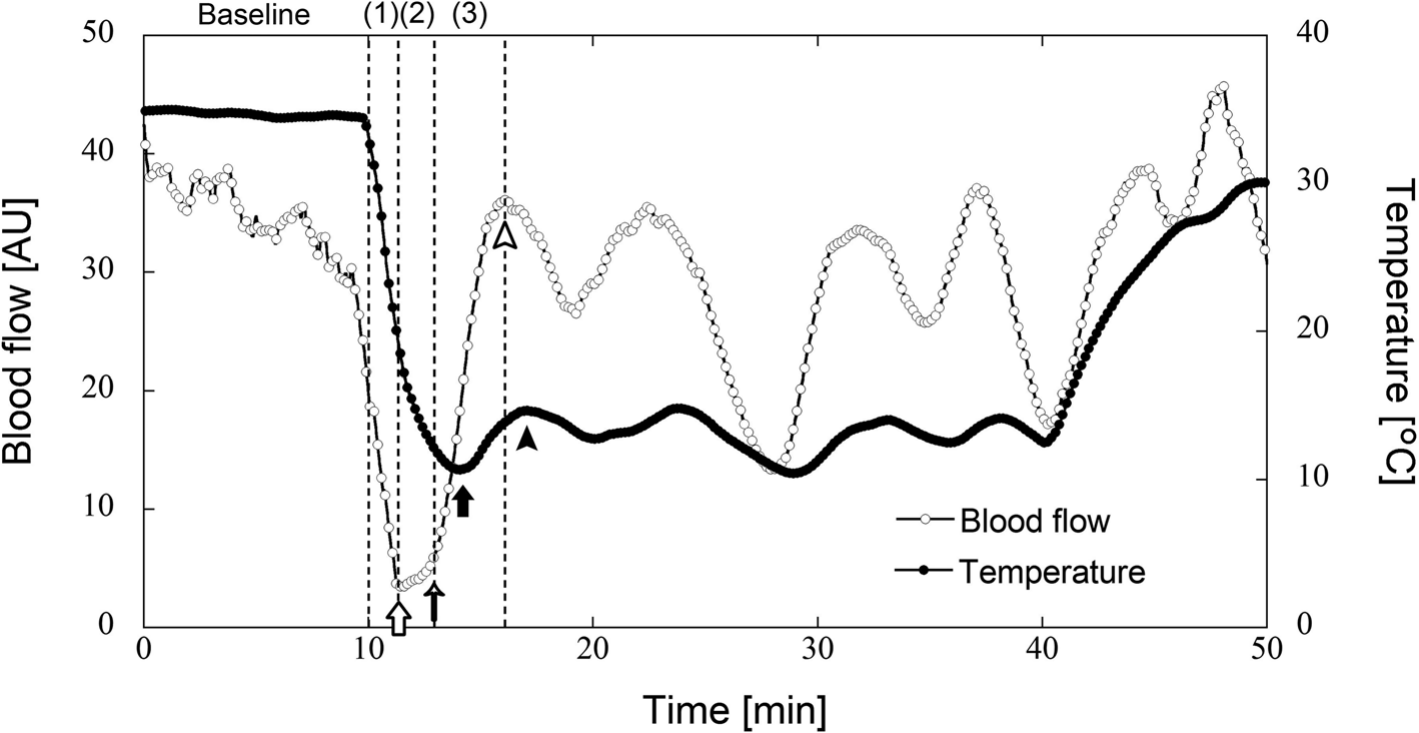
Representative blood flow and temperature in response to finger cooling for individuals with CIVD response at room temperature (25 °C). This figure is comparable to Fig. 1 in our previous study [9]. White and black dots indicate blood flow and temperature, respectively. The finger was exposed to cold water at 5 °C for 30 min following 10 min of rest (Baseline). The first minimum and maximum temperatures are indicated by black arrows and arrowheads, respectively, suggesting the presence of a CIVD event. The first maximum and minimum blood flows are indicated by white arrowheads and arrows, respectively. The onset time of CIVD (CIVD onset_sbf) is indicated by small arrows. Phase 1 (vasoconstriction; from the onset of cooling to minimum blood flow), phase 2 (prior to CIVD; from minimum blood flow to CIVD onset_sbf), and phase 3 (CIVD; from CIVD onset_sbf to maximum blood flow) are indicated in parentheses as 1–3, respectively. AU, arbitrary units

Finger SkBF was sampled at 200 Hz using the MP150 program (BIOPAC Systems, Inc.). Oscillation data were downsampled to 5 Hz using AcqKnowledge software (BIOPAC Systems, Inc.), and a Morlet mother wavelet was applied to conduct wavelet analysis of the SkBF signals using the Biomass program (ELMEC Inc.). The wavelet amplitudes were presented as a ratio of the total power between 0.005 and 0.150 Hz covering the frequency bands related to eNO-independent (0.005–0.010 Hz), eNO-dependent (0.01–0.02 Hz), and neurogenic (0.02–0.05 Hz) activities [5, 8, 12, 13]. We defined a CIVD event according to the definition of Sera et al. [9], which was as follows: “A CIVD event was determined as a minimum increase in finger temperature after the blood flow reached the minimum first, due to local cooling, and then increased.” In other words, followed by the reaction that the blood flow reached the minimum first prior to reascending it, the finger temperature also reached the minimum and then increased. This event was determined as a CIVD. The authors also defined three phases resulting from marked changes in the SkBF during finger cold exposure: vasoconstriction (phase 1), prior to CIVD (phase 2), and CIVD (phase 3). These were defined according to the timing of the increased rate of SkBF due to the onset of the CIVD [9]. Figure 1 shows representative finger SkBF and temperature in response to finger cooling. Phases 1, 2, and 3 represent the periods from the beginning of cold exposure to minimum blood flow, from the minimum blood flow to the onset of CIVD, and from the onset of CIVD to maximum blood flow, respectively. We investigated the wavelet amplitudes at these phases as well as at the baseline. Detailed experimental and data collection methods were described previously by Sera et al. [9].

### Genome-wide genotyping and imputation

An Oragene DNA Kit (DNA Genotek, Ottawa, Canada) was used to collect ≥ 2 mL of saliva from each subject. From these samples, genomic DNA extraction and genotyping were conducted using an Axiom Japonica array (Toshiba, Tokyo, Japan) [14] by Cell Innovator Co., Ltd (Fukuoka, Japan), resulting in ~ 660,000 genetic variants among 94 study subjects, with a < 3% missing rate of the genotype data. Subsequently, we filtered out the genetic variants described below using PLINK ver. 1.90 [15]. After checking gender matching between phenotypic records and heterozygosity of genetic variants on chromosome X and cryptic relatedness between DNA samples (proportion identity with a descent threshold of ≥ 0.1875), we performed quality control (QC) of the genotype data and discarded the following variants: variants with a call rate of < 0.97, variants for which the genotype distribution significantly (*p* < 0.0001) deviated from the Hardy–Weinberg equilibrium, variants with a minor allele frequency (MAF) of < 0.005, and variants on sex chromosomes and the mitochondrial genome. Consequently, 622,446 genetic variants were used for genotype imputation.

After prephasing the genotype data that passed QC via SHAPEIT v2.r904 [16, 17], untyped genotype data were imputed with the 1000 Genomes Project (1KGP) reference panel (phase 3) using IMPUTE2 ver. 2.3.2 [18] (Ne = 20,000; chunk size = 5 Mb). Genetic variants with low imputation quality (info score of < 0.5) were discarded when the imputed output data were converted into PLINK format data using GTOOL ver. 0.7.5 (https://www.well.ox.ac.uk/~cfreeman/software/gwas/gtool.html). We also conducted QC for the imputed genotype data in the same manner, although a 1% MAF threshold was set for the imputed data.

To assess population stratification on a genome-wide scale, we performed principal component analysis (PCA) using the Japonica array genotype data that passed QC in the JPQ cohort after removing genetic variants with a MAF of < 0.05 as well as datasets of five East Asian populations [CDX (Chinese Dai in Xishuangbanna, China), CHB (Han Chinese in Beijing, China), CHS (Southern Han Chinese), JPT (Japanese in Tokyo, Japan), and KHV (Kinh in Ho Chi Minh City, Vietnam)] retrieved from 1KGP [19] phase 3 reference panels (NCBI Build GRCh37; http://bochet.gcc.biostat.washington.edu/beagle/1000_Genomes_phase3_v5a). The datasets of 1KGP populations were generated from variant call format (VCF) data using the PLINK program after the VCF data were converted to the binary VCF data using SAMtools/BCFtools ver. 1.9 [20]. To detect possible population outliers, PCA was also conducted using the genotype data of the JPQ cohort only.

### Construction of a network of CIVD-related categorical data

We focused on two SkBF signals, eNO-independent and neurogenic activities, as these factors can largely affect the finger SkBF response during local cold exposure [9]. Based on the results in our finger cold-water immersion experiment, we categorized the physiological responses of the study subjects in the following manner. First, the subjects were divided into two groups with either the presence or absence of a CIVD response. Second, the subjects were assigned to two additional groups in which they showed either higher or lower amplitudes of each SkBF signal (i.e., eNO-independent and neurogenic activities) at phase 2 compared with the respective mean wavelet amplitudes in individuals who did not show an apparent CIVD response. Consequently, we generated a binary dataset of three categorical variables: (i) the presence or absence of a CIVD response and higher-than-average or lower-than-average wavelet amplitudes of (ii) eNO-independent and (iii) neurogenic activities. Using the dataset, we constructed a network of the three CIVD-related categorical variables using the median-joining method [21] via NETWORK 10.1.0.0 (https://www.fluxus-engineering.com; Fluxus Technology Ltd., Colchester, England).

### Genome-wide association studies of CIVD

We tested the significance of the association between the three aforementioned CIVD-related categorical data types and single-nucleotide polymorphisms (SNPs) using logistic regression analysis and Fisher’s exact test with 1,000,000 max(T) permutations in the additive model via the PLINK program. In addition, two-way analysis of variance (ANOVA) was conducted using CIVD response (presence or absence) and SNP genotypes as independent variables as well as the measurements of SkBF signals related to eNO-independent and neurogenic activities at phase 2, at which stage the amplitudes were remarkably changed [9], as dependent variables. To conduct two-way ANOVA, PLINK format data were converted into the suitable format for the analysis using the Bioconductor R package snpStats ver. 1.20.0 [22] via R ver. 3.4.2 [23]. Furthermore, the genotype data for all subjects were converted into numeric data with dominant and additive genetic inheritance models. Two-way ANOVA was conducted using R ver. 3.5.3 [24] in RStudio ver. 1.2.5042 [25].

SNPs affecting CIVD are expected to be shared between GWASs of phenotypes derived from SkBF signals. Moreover, SkBF is likely controlled by various genetic variants (i.e., polygenic inheritance or epistasis) because quantitative traits related to vascular function and diseases, such as peripheral artery disease and hypertension, can be affected by two or more genes [26–29]; thus, the effect of each SNP on CIVD may not be large. Therefore, we identified candidate CIVD-related genetic variants as follows: SNPs associated with the two CIVD-related SkBF signals or three CIVD-related categorical data (see “Construction of a network of CIVD-related categorical data”) were obtained with a *p* value threshold of < 1 × 10^−5^, which was considered a borderline significant level. We then searched SNPs for which the *p* value was < 5 × 10^−8^ at a genome-wide level in GWASs for either eNO-independent or neurogenic activities. Of the SNPs with *p* values at this significant level, we identified SNPs associated with the other activity at a borderline significant level as candidate CIVD-related genetic variants.

Using a generalized estimating equation (GEE) model [30, 31] via the R package “geepack” [32], we also examined the association between repeated measurements of eNO-independent or neurogenic activities across all phases and combined phenotypes of SNP genotypes with CIVD response (presence or absence). Because the wavelet amplitudes were continuous data, a Gaussian distribution for the family with an identity link was applied to assess the association in the GEE method. The working correlation matrix (correlation of repeated measurements within an individual) was set to a first-order autoregressive model, and the wave argument was used to specify the ordering of repeated measurements within individuals.

Manhattan plots of *p* values were produced using the R package “CMplot” (https://github.com/YinLiLin/R-CMplot). Linkage disequilibrium (LD) between pairs of SNPs in the JPQ cohort was estimated using Haploview version 4.2 [33], whereas the LD in JPT from the 1KGP database was assessed using LDproxy in the LDlink web-based tool (https://ldlink.nci.nih.gov/) [34]. Information on the association of genetic variants with vascular function or disease was searched in the Genome-Wide Repository of Associations Between SNPs and Phenotypes (GRASP, https://grasp.nhlbi.nih.gov/Overview.aspx) [35] and GWAS Catalog (https://www.ebi.ac.uk/gwas/home) [36] databases. To predict the effects of SNPs on protein function, Combined Annotation Dependent Depletion (CADD) scores (https://cadd.gs.washington.edu/) [37] were used. The relevant biological pathways of genes containing focal SNPs were estimated using the Kyoto Encyclopedia of Genes and Genomes (KEGG) database (https://www.genome.jp/kegg/).

## Results

### Physiological responses to cold-water immersion

**Fig. 2:**
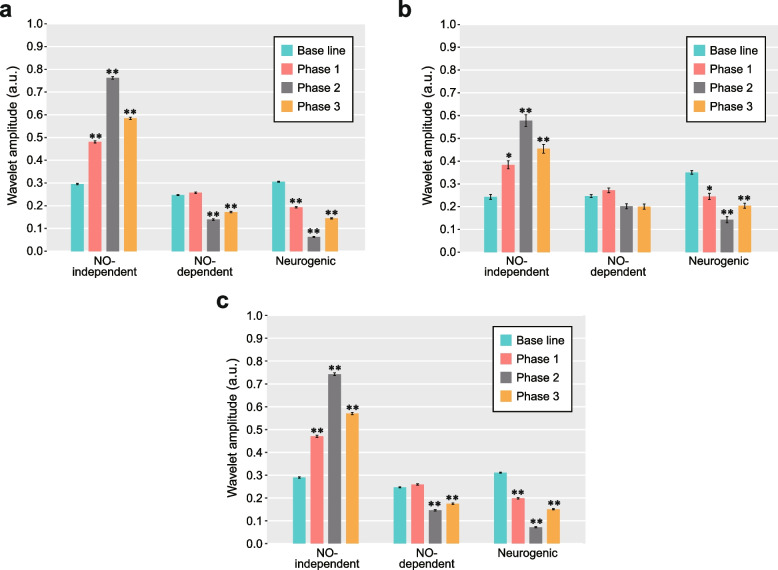
Wavelet amplitudes of endothelial NO-independent, endothelial NO-dependent, and neurogenic activities in four phases, including the baseline phase, in the study subjects. These subjects showed the presence (**a**) or absence (**b**) of a CIVD response (**a** *n* = 84; **b** *n* = 10). All study subjects (*n* = 94) are shown in **c**. Phases 1, 2, and 3 represent “vasoconstriction,” “prior to CIVD,” and “CIVD,” respectively [9]. Data are means ± standard error of the mean. * *p* < 0.05 and ** *p* < 0.01 (compared with the baseline using Dunnett’s test). a.u., arbitrary units

**Fig. 3:**
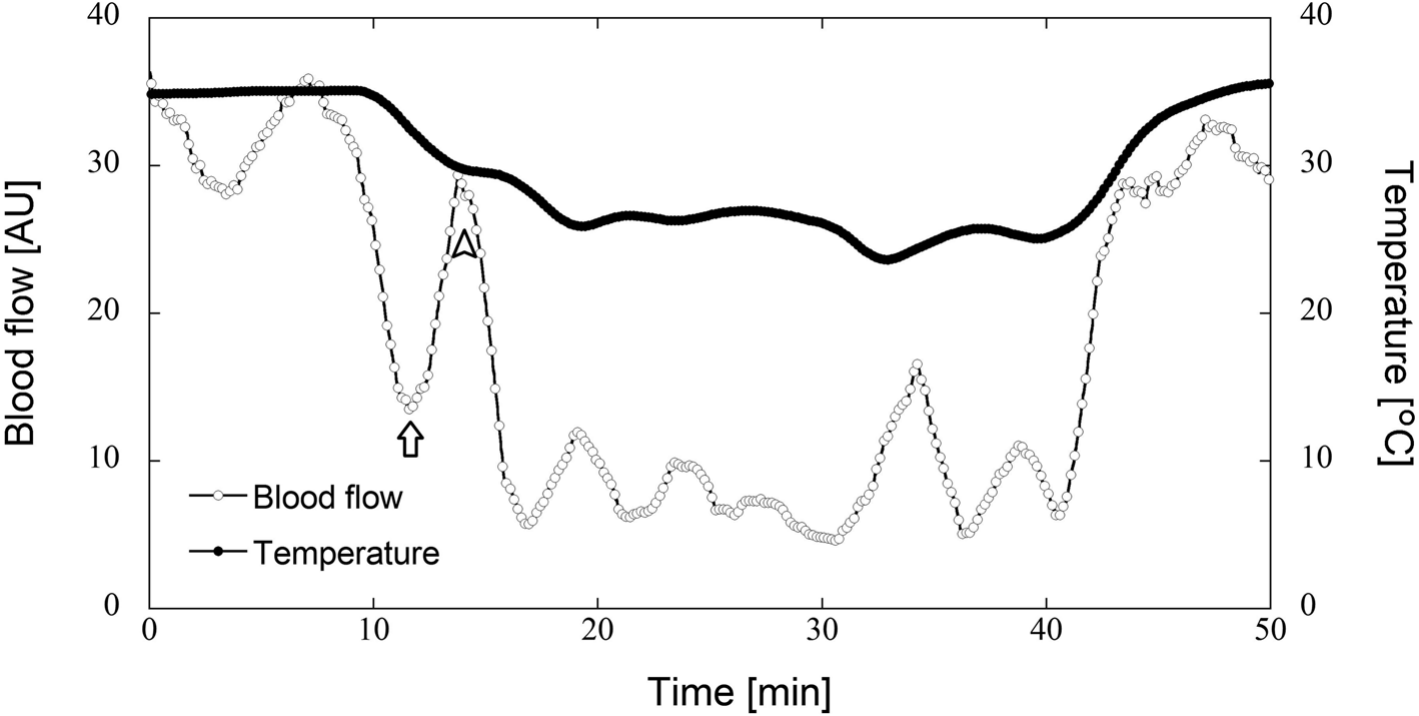
Representative blood flow and temperature in response to finger cooling for individuals without CIVD response at room temperature (25 °C). White and black dots indicate blood flow and temperature, respectively. The finger was exposed to cold water at 5 °C for 30 min following 10 min of rest (Baseline). The first maximum and minimum blood flows are indicated by white arrowheads and arrows, respectively. AU., arbitrary units

In total, 94 healthy Japanese volunteers (93 men and 1 woman), referred to here as the “JPQ cohort,” were recruited from Kyushu University. The characteristics of the 94 subjects in this cohort are summarized in Table 1. In the finger cold-water immersion experiment (see “Methods”), wavelet analyses of SkBF signals were conducted to quantify the activities of the following vascular regulatory factors: eNO-independent, eNO-dependent, and neurogenic activities. Interestingly, no apparent CIVD response was observed in 10 subjects (~ 10%), whereas the remaining 84 subjects exhibited at least one CIVD response. In the 84 subjects with CIVD responses, the patterns of wavelet amplitudes across the four phases [baseline, vasoconstriction (phase 1), prior to CIVD (phase 2), and CIVD (phase 3); Fig. 2a] were in agreement with those reported in our previous study [9]. The amplitudes of eNO-independent activity increased until phase 2, after which they decreased; conversely, the amplitudes of neurogenic activity decreased until phase 2, after which they increased. The differences in the wavelet amplitudes of eNO-independent and neurogenic activities between the baseline and phases 1–3 were statistically significant (*p* < 0.05, Dunnett’s test). For eNO-dependent activity, the amplitudes between the baseline and phase 1 did not differ significantly (*p* = 0.734), whereas the amplitudes between the baseline and phases 2 and 3 differed significantly (*p* < 0.0001). The representative patterns of finger SkBF and temperature in response to finger cooling for individuals with CIVD response and without apparent CIVD response are shown in Figs. 1 and 3, respectively.

**Table 1:** Characteristics of study subjects with or without CIVD response

Characteristic	NonCIVD	CIVD
No. of subjects	10	84
Age, years	22.80 ± 0.44	22.24 ± 0.15
Height, cm	171.30 ± 2.30	171.83 ± 0.60
Weight, kg	62.22 ± 2.73	64.25 ± 1.20
Body mass index, kg/m^2^	21.24 ± 0.93	21.73 ± 0.38
Body temperature, °C	36.61 ± 0.13	36.45 ± 0.04
Minimum finger temperature during local cooling, °C	12.9 ± 2.0	10.3 ± 0.4
Mean finger temperature between 15 and 35 min, °C	16.6 ± 2.3	13.6 ± 0.4
Time for the first minimum blood flow, min^a^	11.9 ± 0.3	11.7 ± 0.1
Neurogenic activity^b^ (Baseline)	0.35 ± 0.03	0.31 ± 0.01
Neurogenic activity^b^ (Phase 1)^c^	0.24 ± 0.05	0.19 ± 0.01
Neurogenic activity^b^ (Phase 2)^c^	0.14 ± 0.05	0.06 ± 0.01
Neurogenic activity^b^ (Phase 3)^c^	0.20 ± 0.04	0.14 ± 0.01
eNO-dependent activity ^b^ (Baseline)	0.25 ± 0.03	0.25 ± 0.01
eNO-dependent activity ^b^ (Phase 1) ^c^	0.27 ± 0.04	0.26 ± 0.01
eNO-dependent activity ^b^ (Phase 2) ^c^	0.20 ± 0.04	0.14 ± 0.01
eNO-dependent activity ^b^ (Phase 3) ^c^	0.20 ± 0.04	0.17 ± 0.01
eNO-independent activity ^b^ (Baseline)	0.24 ± 0.04	0.30 ± 0.01
eNO-independent activity ^b^ (Phase 1) ^c^	0.38 ± 0.07	0.48 ± 0.02
eNO-independent activity ^b^ (Phase 2) ^c^	0.58 ± 0.10	0.76 ± 0.02
eNO-independent activity ^b^ (Phase 3) ^c^	0.45 ± 0.08	0.58 ± 0.02

Data are means ± standard error of the mean

*Abbreviations: CIVD* Cold-induced vasodilation, *eNO* endothelial nitric oxide

^a^Time corresponding to the white arrow in Figs. 1 and 3

^b^Wavelet amplitude estimated from finger skin blood flow (arbitrary units)

^c^Phases 1, 2, and 3 represent “vasoconstriction,” “prior to CIVD,” and “CIVD,” respectively [9]

In the 10 subjects who did not exhibit a CIVD response, there were no significant differences in eNO-dependent activity between the baseline and phases 1–3 (*p* = 0.6674–0.9149), but eNO-independent and neurogenic activities between the baseline and phase 2 or 3 (*p* $\le$ 0.0018) differed significantly (Fig. 2b). We postulate that eNO-dependent activity is not a major factor in the CIVD response because the mean values of the amplitude at phase 2 were lower than those at the baseline (Fig. 2). In addition, the decreased activity of eNO, which is a vasodilator, at phase 2 is likely related to reduced vasodilation, which would indicate that eNO-dependent activity does not primarily influence CIVD. The wavelet amplitudes of each activity in all study subjects were shown in Fig. 2c. Overall, wavelet analysis indicated that eNO-independent and neurogenic activities potentially contribute to the CIVD response.

### Genotype data in the JPQ cohort

**Fig. 4:**
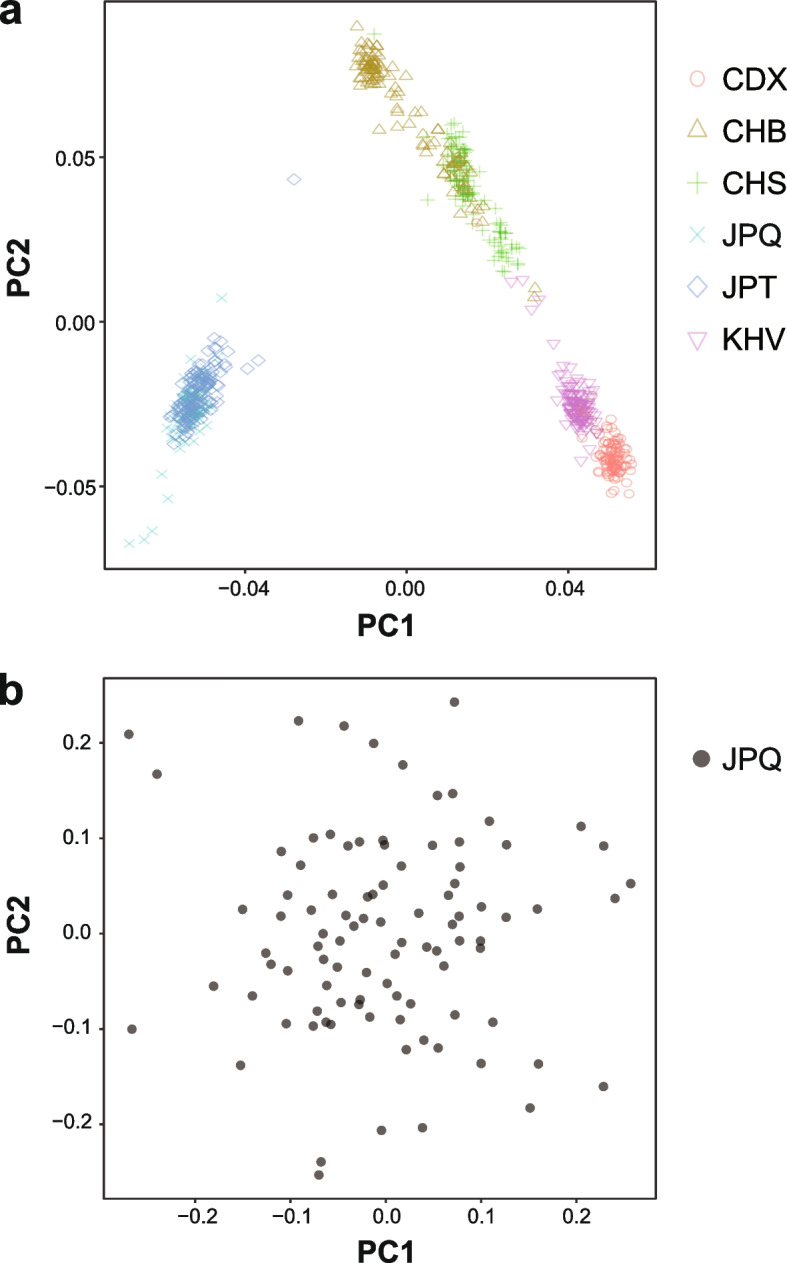
Principal component analysis of genotype data from East Asian populations (**a**) and the JPQ cohort (**b**). Data were plotted according to the first (horizontal axis) and second (vertical axis) principal components. CDX: Chinese Dai in Xishuangbanna, China; CHB: Han Chinese in Beijing, China; CHS: Southern Han Chinese; JPQ: Japanese in Kyushu, Japan; JPT: Japanese in Tokyo, Japan; KHV: Kinh in Ho Chi Minh City, Vietnam

In the JPQ cohort, ~ 660,000 genetic variants among the 94 subjects were genotyped using an Axiom Japonica array. Following QC of the genotype data, 622,446 genetic variants were generated. Population stratification was assessed on a genome-wide scale using the genotype datasets of the JPQ cohort as well as five East Asian populations (CDX, CHB, CHS, JPT, and KHV) from the 1KGP database after removing genetic variants with a MAF of < 0.05 (Fig. 4a). A PCA plot showed that the eigenvectors of the JPQ cohort and JPT (consisting of Japanese individuals) formed a distinct cluster from those of the other East Asian populations. The JPQ genotype data were also used in isolation to examine population outliers in the PCA, and such outliers were not detected in this analysis (Fig. 4b); thus, we used all JPQ cohort genotype data for genotype imputation. After imputation and QC of the genotype data (see “Methods” for the detailed methods of both), 4,661,825 SNPs among the 94 Japanese subjects were used for GWASs of CIVD-related traits.

### Association of SNPs with CIVD-related traits in the JPQ cohort

In GWASs, we focused on eNO-independent and neurogenic activities because these factors likely affect the CIVD response. However, in GWASs in which logistic regression analysis and Fisher’s exact test were conducted using the three CIVD-related categorical data, there was a lack of SNPs associated with empirical *p* values of < 5 × 10^−8^ at the genome-wide significant level although 187 and 71 genetic variants showing a suggestive association (*p* < 1 × 10^−5^) were detected in the logistic regression analysis and Fisher’s exact test, respectively (Additional file 1: Tables S2 − 3).

**Fig. 5:**
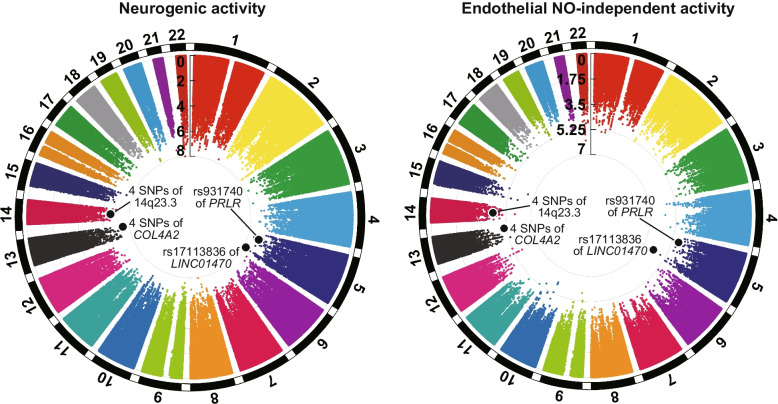
Manhattan plots of *p* values [plotted as − log_10_(*p*)] in genome-wide association studies of CIVD responses with the additive model

Next, we conducted two-way ANOVA for the GWASs of eNO-independent and neurogenic activities in the additive and dominant genetic models to test for associations between these activities and the interaction between CIVD response and SNPs. Manhattan plots of GWASs for eNO-independent and neurogenic activities are shown in Fig. 5. We detected 10 SNPs associated with CIVD-related phenotypes (Table 2 and Additional file 1: Tables S4 − 6). The GWASs with two-way ANOVA revealed that interactions between CIVD response and five SNPs [rs17113836 of *LINC01470* (long intergenic nonprotein coding RNA 1470) or four SNPs of *COL4A2* (collagen type IV alpha 2 chain)] were associated with neurogenic activity at phase 2 at the genome-wide significant level (*p* value range 1.89 × 10^−8^ to 3.24 × 10^−8^) in the additive model (Table 2 and Additional file 1: Fig. S1a). These SNPs were also associated with eNO-independent activity at phase 2 at a borderline significant level (*p* value range 7.02 × 10^−7^ to 9.36 × 10^−6^). In the dominant model, five SNPs [rs931740 of *PRLR* (prolactin receptor) and four SNPs at 14q23.3] met the criteria mentioned in “Methods” (*p* value ranges 1.03 × 10^−8^ to 4.78 × 10^−8^ for neurogenic activity; 3.29 × 10^−6^ to 9.39 × 10^−6^ for eNO-independent activity) (Table 2 and Additional file 1: Fig. S1b).

**Table 2:** Summary of SNPs showing the significant association of CIVD with neurogenic and endothelial NO-independent activities according to two-way ANOVA

Genetic model	RefSNP ID	Position^a^	Gene	Allele frequency	CADD score	Neurogenic		eNO-independent	
						*F*	*P*	*F*	*P*
Additive	rs17113836	5: 152,344,712	*LINC01470*	A: 0.72 T: 0.28	0.3	38.3	1.89 × 10^−8^	28.6	7.02 × 10^−7^
	rs73619297	13: 110,967,247	*COL4A2*	G: 0.62 A: 0.38	0.7	21.1	3.24 × 10^−8^	13.2	9.36 × 10^−6^
	rs73619299	13: 110,967,456	*COL4A2*	C: 0.62 T: 0.38	2.8	21.1	3.24 × 10^−8^	13.2	9.36 × 10^−6^
	rs7321461	13: 110,967,990	*COL4A2*	A: 0.62 C: 0.38	0.1	21.1	3.24 × 10^−8^	13.2	9.36 × 10^−6^
	rs3832899	13: 110,968,318	*COL4A2*	T: 0.62 –: 0.38	1.5	21.1	3.24 × 10^−8^	13.2	9.36 × 10^−6^
Dominant	rs931740	5: 35,222,040	*PRLR*	T: 0.59 C: 0.41	5.3	39.9	1.03 × 10^−8^	24.6	3.29 × 10^−6^
	rs10144241	14: 65,638,439	14q23.3	C: 0.57 A: 0.43	11.3	35.6	4.78 × 10^−8^	22.1	9.39 × 10^−6^
	rs10133408	14: 65,638,443	14q23.3	T: 0.57 G: 0.43	14.1	35.6	4.78 × 10^−8^	22.1	9.39 × 10^−6^
	rs11850650	14: 65,639,582	14q23.3	T: 0.57 C: 0.43	7.4	35.6	4.78 × 10^−8^	22.1	9.39 × 10^−6^
	rs11158583	14: 65,641,686	14q23.3	G: 0.57 C: 0.43	9.3	35.6	4.78 × 10^−8^	22.1	9.39 × 10^−6^

*Abbreviations: CADD* Combined Annotation Dependent Depletion, *CIVD* Cold-induced vasodilation, *eNO* endothelial nitric oxide, *GWAS* Genome-wide association study, *SNP* Single-nucleotide polymorphism

^a^Chromosomal position in NCBI build GRCh37

**Fig. 6:**
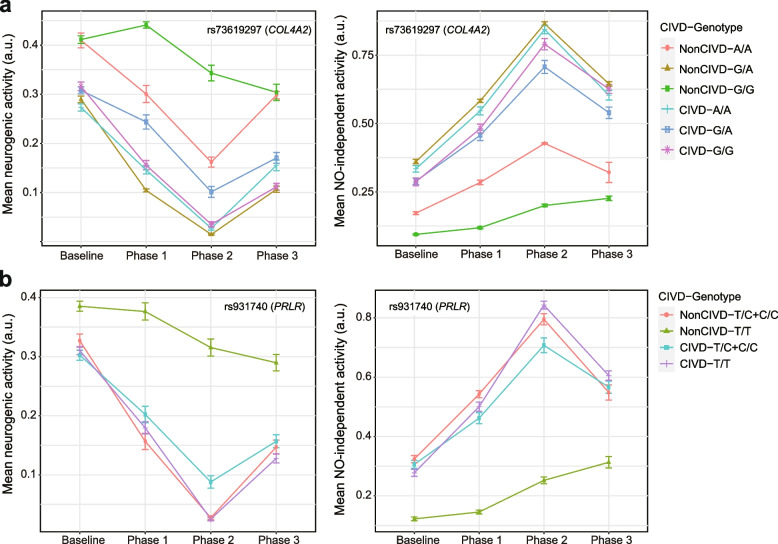
Wavelet amplitudes of neurogenic and endothelial NO-independent activities across four phases, including the baseline phase, in groups of subjects (*n* = 94). These subjects were differentiated by CIVD response and the genotypes of rs73619297 of *COL4A2* (**a**) or rs931740 of *PRLR* (**b**). Phases 1, 2, and 3 represent “vasoconstriction,” “prior to CIVD,” and “CIVD,” respectively [9]. Data are means ± standard error of the mean. a.u., arbitrary units

We examined the wavelet amplitudes of eNO-independent and neurogenic activities across all phases (repeated measurements) in groups of subjects differentiated by CIVD response and rs73619297 of *COL4A2* (Fig. 6a) or rs931740 of *PRLR* (Fig. 6b). Interestingly, the homozygotes of the major alleles in subjects who had no apparent CIVD response (NonCIVD–G/G for rs73619297 and NonCIVD–T/T for rs931740) had the lowest mean amplitudes of eNO-independent activity across the phases, whereas the mean amplitudes of neurogenic activity in these homozygotes were consistently the highest amplitudes. Using a GEE model, we also tested the significance of differences between the repeated measurement data of wavelet amplitudes in subjects with the NonCIVD–G/G or NonCIVD–T/T and other CIVD-genotype categories for each SNP. To minimize effects of possible population stratification, we used PC1 and PC2 estimated from PCA analysis based on the imputed data (Additional file 1: Fig. S2) as covariates. GEE analyses with adjustment for PC1 and PC2 indicated that, in the additive model, the eNO-independent activity of subjects with the NonCIVD–G/G for rs73619297 differed significantly from that of the remaining subjects (*p* = 0.0056), whereas such a difference was not detected for neurogenic activity (*p* = 0.1955). The eNO-independent and neurogenic activities differed significantly between subjects with NonCIVD–T/T for rs931740 and the remaining subjects in the dominant model (*p* = 7.3 × 10^−5^ for eNO-independent activity;* p* = 0.0082 for neurogenic activity).

### Network analysis of CIVD-related categorical data

**Fig. 7:**
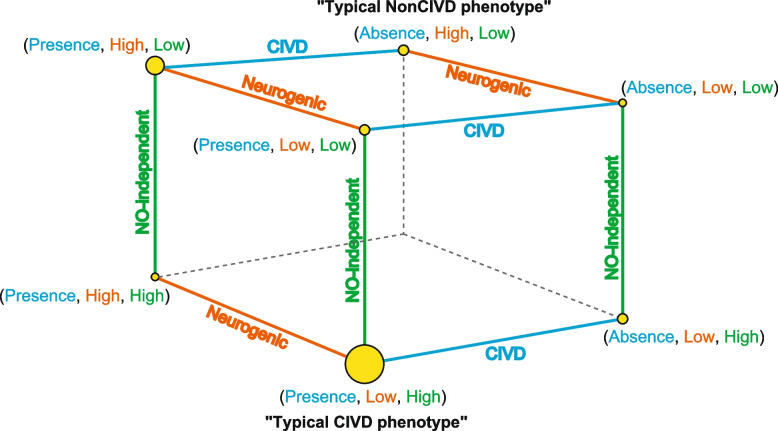
Network of CIVD-related factors (CIVD response, endothelial NO-independent activity, and neurogenic activity). “Presence” and “Absence” indicate the presence and absence of CIVD response, respectively. “Low” and “High” indicate lower-than-average and higher-than-average wavelet amplitudes, respectively, of endothelial NO-independent or neurogenic activities at phase 2. “Typical CIVD phenotype” group consisted of individuals with a CIVD response, lower-than-average neurogenic activity, and higher-than-average eNO-independent activity. “Typical NonCIVD phenotype” group consisted of individuals without a CIVD response, higher-than-average neurogenic activity, and lower-than-average eNO-independent activity

Classification according to CIVD response or wavelet amplitudes of eNO-independent and neurogenic activities at phase 2 (see “Methods”) assigned study subjects to 20 and 74 subjects with lower and higher eNO-independent activities, respectively, and 78 and 16 subjects with lower and higher neurogenic activities, respectively. We then constructed a network of categorical groups to visualize the relationships among the three CIVD-related factors (Fig. 7). Network analysis displayed that the study subjects could be assigned to seven groups. The main group consisted of 72 individuals who exhibited a CIVD response, had higher-than-average eNO-independent activity, and had lower-than-average neurogenic activity. The characteristics related to this group are referred to hereafter as the “typical CIVD phenotype.” Contrastingly, four individuals showed the opposite pattern to that in the main group, i.e., the absence of a CIVD response, lower-than-average eNO-independent activity, and higher-than-average neurogenic activity. The characteristics of this group are referred to as the “typical NonCIVD phenotype” (Fig. 7).

We compared the relationships between the genotypes of the 10 candidate SNPs and the network of categorical data (Additional file 1: Tables S7 and S8). It was difficult to define the 72 subjects with the typical CIVD phenotype by the genotypes of candidate SNPs only. However, with the exception of one individual, all subjects with the typical NonCIVD phenotype were homozygotes of major alleles at the candidate SNPs (Additional file 1: Tables S7 and S8). As mentioned, the amplitudes of eNO-independent and neurogenic activities in subjects with NonCIVD–T/T for rs931740 and NonCIVD–G/G for rs73619297 remained low and high, respectively, across all phases relative to the amplitudes in the other CIVD-genotype categorical groups (Fig. 6). Thus, their major alleles may partly affect the blunted response of eNO-independent and neurogenic activities.

In the 10 subjects without a CIVD response, there was less fluctuation in the wavelet amplitudes of eNO-independent and neurogenic activities from the baseline to phase 3 in the 4 subjects with the typical NonCIVD phenotype relative to the corresponding amplitudes in the other subjects (Additional file 1: Fig. S3). This suggests that the response of skin blood vessels (i.e., vasoconstriction or vasodilation) in these four subjects is less susceptible to cold stimuli. We hypothesized that the blunted response of blood vessels resulted in the maintenance of skin temperature and tested this hypothesis by examining the minimum skin temperature of study subjects in a cold-water immersion experiment. The mean values of minimum skin temperature in subjects with the presence or absence of a CIVD response were 10.3 °C ± 0.4 °C and 12.9 °C ± 2.0 °C, respectively. These values are the minimum finger temperatures in response to finger cooling, and thereafter, the temperature increases as the blood flow increases when CIVD is occurred (Fig. 1). In the four individuals with the typical NonCIVD phenotype, the mean value of minimum skin temperature, 18.0 °C ± 3.3 °C, was substantially higher than that in the other individuals. The results suggest that the sensitivity of vasoconstriction and vasodilation to local cold stress are low in individuals with the typical NonCIVD phenotype because they can maintain the minimum skin temperature at relatively high levels, compared to individuals with the other CIVD-related phenotypes, during finger cold-water immersion.

### Linkage disequilibrium analysis and functional prediction of candidate genes

We surveyed the LD between the candidate SNPs and their neighboring SNPs using LDproxy in the LDlink database. According to this database, the candidate SNPs were in LD with several SNPs in JPT (*r*^2^ > 0.8; Additional file 1: Table S9): Two SNPs and one SNP were in LD with rs931740 of *PRLR* and rs17113836 of *LINC01470*, respectively, whereas rs73619297 of *COL4A2* and rs10144241 at 14q23.3 were in LD with six and twenty-three SNPs, respectively. The candidate SNPs and the adjacent SNPs, which were in LD with the query SNPs, were not located in the exonic region. In the JPQ cohort, each of the four candidate SNPs in *COL4A2* and 14q23.3 were also in strong LD (D´ = 1.00; *r*^2^ = 1.00; Additional file 1: Fig. S4). These results suggest that the associations of the focal SNPs in *COL4A2* or 14q23.3 with the CIVD-related traits are not independent. According to the GWAS Catalog and GRASP databases, no SNPs were significantly associated with vascular function or disease. Therefore, it remains difficult to determine the SNPs that are responsible for the CIVD response.

We also examined the CADD-scaled C-scores of candidate SNPs associated with CIVD to predict the effects of the SNPs on protein function (Table 2). The candidate SNPs had scores < 15 (CADD-scaled C-score range 0.1–14.1), and the scores of *PRLR* and *COL4A2* SNPs were low (CADD-scaled C-score range 0.1–5.3), suggesting that the focal SNPs do not have strong effects on protein function.

Finally, we predicted the biological pathways of *PRLR* and *COL4A2* using the KEGG database. Accordingly, *PRLR* and *COL4A2* were involved in 5 and 10 KEGG pathways, respectively (Table 3). KEGG analysis also indicated that these genes were involved in the PI3K/Akt signaling pathway [38, 39], which plays important roles in vascular homeostasis and angiogenesis [40].

**Table 3:** KEGG pathways of *PRLR* and *COL4A2*

Gene name	KEGG ID	Pathway
*PRLR*	hsa04060	Cytokine-cytokine receptor interaction
	hsa04080	Neuroactive ligand-receptor interaction
	hsa04151	PI3K-Akt signaling pathway
	hsa04630	JAK-STAT signaling pathway
	hsa04917	Prolactin signaling pathway
*COL4A2*	hsa04151	PI3K-Akt signaling pathway
	hsa04510	Focal adhesion
	hsa04512	ECM-receptor interaction
	hsa04926	Relaxin signaling pathway
	hsa04933	AGE-RAGE signaling pathway in diabetic complications
	hsa04974	Protein digestion and absorption
	hsa05146	Amoebiasis
	hsa05165	Human papillomavirus infection
	hsa05200	Pathways in cancer
	hsa05222	Small cell lung cancer

## Discussion

In our previous study, we found that the eNO-independent activity in 18 subjects significantly increased at vasoconstriction (phase 1) and prior to CIVD (phase 2) [9]. To verify this finding, we performed the replication study using a larger sample size (*n* = 94). In a finger cold-water immersion experiment, we replicated the significant increase in eNO-independent activity at phases 1 and 2. We also replicated the significant decrease in neurogenic activity at these phases, as was observed in the previous study [9]. These results indicate that eNO-independent and neurogenic activities contribute to the CIVD response during finger cold exposure. Furthermore, we found that 10 of 94 study subjects had no apparent CIVD response.

In humans, NO reportedly plays an important role in the dilation of skin blood vessels induced by local warming of the skin [41–43]. However, the cold-water immersion experiments in our previous [9] and present studies indicated that eNO-dependent activity may not have a substantial effect on the CIVD response. Given that NO-dependent vasodilation may act primarily on the superior mesenteric arteries (~ 650 µm in diameter) in rats whereas NO-independent vasodilation may be relevant to small arteries, e.g., resistance arteries (~ 200 µm in diameter) [10], we previously hypothesized that CIVD is largely unaffected by eNO-dependent activity [9] because the inner diameter of human arteriovenous anastomoses (AVAs), which may play an important role in CIVD, is relatively small (~ 10–150 µm) [44]. Although a diameter of capillaries is smaller than that of AVAs, capillary generally consists of a thin wall of endothelial cells and the contribution to blood flow regulation is lower. Therefore, the relaxation of AVAs by eNO-independent activity, rather than eNO-dependent activity, is more likely to contribute to CIVD.

In the present study, we detected 10 candidate SNPs in GWASs of CIVD using two-way ANOVA. These SNPs were not shared with genetic variants moderately associated with three CIVD-related categorical data in logistic regression analyses or Fisher’s exact test (*p* < 1 × 10^−5^). This inconsistency might be due to the difference of statistical method used. In addition, given that the effect of each SNP on CIVD is not large, larger sample size which can increase statistical power is required to detect a more definitive association between SNPs and CIVD response. One and four of the 10 candidate SNPs identified by the GWASs with two-way ANOVA are located in *PRLR* and *COL4A2*, respectively, whereas rs17113836 is in *LINC01470*. Because *LINC01470* is not well characterized, the functional relevance of this long noncoding RNA to CIVD could not be inferred. Regarding the SNPs of the functional genes, we found that the study subjects with NonCIVD–T/T for *PRLR* rs931740 or NonCIVD–G/G for *COL4A2* rs73619297 showed blunted changes in eNO-independent and neurogenic activities during finger cold exposure. Notably, the main group also contained the homozygotes of the major alleles of rs931740 and rs73619297. Thus, we can hypothesize that the major alleles of *PRLR* and *COL4A2* SNPs have effects in concert with other genetic variants across the genome on the blunted CIVD-related activities, although the individual effects of each SNP were not large. Alternatively, epigenetics might drive the phenotypic plasticity in response to local cold exposure. Functional analysis or an epigenome-wide association study will help validate these results.

*PRLR* encodes a receptor belonging to a type I cytokine receptor superfamily that binds prolactin, which is an anterior pituitary hormone. Prolactin and its receptor (PRLR) can regulate a wide variety of physiological functions in the nervous system [45]. Additionally, prolactin can suppress the expression of inducible NO synthase (NOS) induced by proinflammatory cytokines [46]. According to our KEGG analysis, *PRLR* is involved in the JAK/STAT signaling pathway, which is known to be induced by JAK2 via a prolactin–PRLR interaction [47, 48]. In addition, the JAK2/STAT3 signaling pathway can regulate tumor angiogenesis through modulation of several angiogenesis-related genes [49, 50]. Given that the interaction between prolactin and PRLR molecules affects regulation of NO-independent vasodilation in rats [51], *PRLR* might contribute to the development and vasodilation of new or existing vessels in response to finger cold exposure.

*COL4A2* encodes the alpha-2 chain of type IV collagen (collagen IV), which is a main component of the basement membrane (BM) and essential for BM stability [52]. *Col4a1*/*Col4a2* knockout mice reportedly die due to various deficiencies, including neuronal ectopias and misarrangement of the capillary network during angiogenesis [52, 53]. Given that (i) the scaffolding of components, including collagen IV, in the BM plays a key role in vascular homeostasis and development [54] and (ii) familial mutations of *COL4A2* can be risk factor for cerebral abnormalities, such as porencephaly and small-vessel disease [55], the four candidate SNPs of *COL4A2* identified in the present study might affect vascular dynamics during finger cold-water immersion through changes in the affinity for COL4A1 (collagen type IV alpha 1 chain) in heterotrimer formation.

According to a KEGG analysis, *PRLR* and *COL4A2* are related to the PI3K/Akt signaling pathway [38, 39], which is known to promote production of NO, a vasodilator, through phosphorylation of endothelial NOS in endothelial cells [40, 56, 57]. However, NO is a potent vasodilator of relatively large vessels [10, 11], and eNO-dependent vasodilation may not be a key factor in the CIVD response [9]. Endothelium-derived hydrogen peroxide has been reported as an EDHF that mediates vasodilation of small resistance vessels in humans [58, 59], and EDHF-mediated relaxation in response to acetylcholine can occur in NOSs (neuronal, inducible, and endothelial NOSs) in a system-dependent manner [60]. Although the relationship between the PI3K/Akt signaling pathway and EDHF has yet to be elucidated, this pathway could potentially be involved in not only NO-dependent vasodilation but also EDHF-mediated vascular relaxation [61, 62]. Thus, we can hypothesize that *PRLR* and *COL4A2* contribute to CIVD through the relaxation of vascular smooth muscle cells mediated by EDHF [11]. Although further studies may reveal the relationship between EDHF-mediated vasodilation and CIVD, we cannot currently rule out the possibility that *PRLR* and *COL4A2* participate in novel CIVD-related biological pathways.

We acknowledge several limitations in the present study. First, the sample size is not sufficiently large to show a significant association between SNPs and CIVD-related traits with high statistical power. This was due to the difficulty in sampling from a large number of subjects for GWASs; however, our study, including a finger cold-water immersion experiment, had the largest sample size in the CIVD research field to date. To validate the candidate genetic variants related to CIVD identified in our GWASs, replication GWASs in other Japanese cohorts or other ethnic groups are required. Second, the functional relevance of the candidate SNPs or genes in relation to CIVD remains unclear. Lastly, the candidate SNPs identified could be in LD with neighboring genetic variants; thus, we cannot rule out the possibility that such neighboring genetic variants may have effects on CIVD. To address these issues, functional analysis using genetic engineering technologies, such as genome editing, should be used to examine the effects of SNPs or genes on phenotypes.

## Conclusions

Our finger cold-water immersion experiment revealed that ~ 10% of the 94 Japanese study subjects did not show an apparent CIVD response. Furthermore, the GWASs of CIVD identified 10 candidate genetic variants associated with CIVD-related traits in the subjects, and two candidate functional genes, *PRLR* and *COL4A2*, were likely involved in vascular function. Our findings suggest that the focal SNPs may affect the blunted SkBF response associated with eNO-independent and neurogenic activities, although SNP with a large effect on CIVD has yet to be detected. It will be worthwhile investigating whether *PRLR* and *COL4A2* are related to EDHF-mediated vasodilation in peripheral tissues. Our results suggest that genetic polymorphisms are attributable to the interethnic variability of the CIVD response through differences in the allelic frequencies of SNPs associated with CIVD. Using the largest-scale dataset of wavelet analysis produced to date, we are the first to report GWASs for CIVD; thus, this study improves our understanding of the mechanisms underlying CIVD.

## Supplementary Information

**Additional file 1:** **Fig. S1**. Wavelet amplitudes of neurogenic and endothelial NO-independent activities at phase 2 (prior to CIVD) in groups of subjects differentiated by their CIVD response and genotypes of (a) rs73619297 of *COL4A2 *or (b) rs931740 of *PRLR*. Data are means ± standard error of the mean. a.u., arbitrary units. **Fig. S2.** Principal component analysis of imputed genotype data from the JPQ cohort. **Fig. S3.** Wavelet amplitudes of endothelial NO-independent, endothelial NO-dependent, and neurogenic activities in four phases, including the baseline phase, in 10 study subjects with no CIVD response. Phases 1, 2, and 3 represent “vasoconstriction,” “prior to CIVD,” and “CIVD,” respectively [1]. a.u., arbitrary units. **Fig. S4.** Physical position and linkage disequilibrium of candidate SNPs around *COL4A2 *or at 14q23.3 in 94 Japanese subjects. The diagram was created using Haploview version 4.2. The numbers in diamonds represent *r*^2^ values (×100). The haplotype block was defined using the method of Gabriel et al. [2]. SNPs shown in bold are CIVD-associated SNPs. **Table S1. **Characteristics of all study subjects including one female. **Table S2.** SNP with *p *< 1×10^-5^ for CIVD-related categorical traits (CIVD response and eNO-independent activity, neurogenic activity) in the logistic regression analysis with the additive model. **Table S3.** SNP with *p *< 1×10^-5^ for CIVD-related categorical traits (CIVD response and eNO-independent activity, neurogenic activity) in the Fisher's exact test with the additive model. **Table S4.** Allele frequency of candidate SNPs detected in GWASs of CIVD response with neurogenic and endothelial NO-independent activities. **Table S5.** SNP with *p *< 1×10^-5^ for eNO-independent and neurogenic activities in the additive model using two-way ANOVA. **Table S6.** SNP with *p *< 1×10^-5^ for eNO-independent and neurogenic activities in the dominant model using two-way ANOVA. **Table S7.** Relationships among the genotypes of five candidate SNPs defined in the dominant model and network of three CIVD-related factors.** Table S8.** Relationships among the genotypes of five candidate SNPs defined in the additive model and network of three CIVD-related factors. **Table S9.** Correlation estimates of four candidate SNPs with their adjacent SNPs using LDproxy.

## Data Availability

Scientifically motivated request for data sharing will be considered. If justified, applicable parts of the data can be made available in anonymized format.

## Abbreviations

ANOVA: Analysis of variance
AVA: Arteriovenous anastomose
BM: Basement membrane
CADD: Combined Annotation Dependent Depletion
CDX: Chinese Dai in Xishuangbanna, China
CHB: Han Chinese in Beijing, China
CHS: Southern Han Chinese
CIVD: Cold-induced vasodilation
COL4A1: Collagen type IV alpha 1 chain
COL4A2: Collagen type IV alpha 2 chain
eNO: Endothelial nitric oxide
EDHF: Endothelium-derived hyperpolarizing factor
GEE: Generalized estimating equation
GRASP: Genome-Wide Repository of Associations Between SNPs and Phenotypes
GWAS: Genome-wide association study
JPT: Japanese in Tokyo, Japan
KEGG: Kyoto Encyclopedia of Genes and Genomes
KHV: Kinh in Ho Chi Minh City, Vietnam
LD: Linkage disequilibrium
LINC01470: Long intergenic nonprotein coding RNA 1470
MAF: Minor allele frequency
NO: Nitric oxide
NOS: NO synthase
PCA: Principal component analysis
PRLR: Prolactin receptor
QC: Quality control
SkBF: Skin blood flow
SNP: Single-nucleotide polymorphism
VCF: Variant call format
1KGP: 1000 Genomes Project
