# Supplementary material for: Cold-induced vasodilation response in a Japanese cohort: insights from cold-water immersion and genome-wide association studies
Source: J Physiol Anthropol. 2023 Mar 8;42:2. doi: 10.1186/s40101-023-00319-2 (PMC9993636; doi:10.1186/s40101-023-00319-2)
Supplement: Supplementary file 1 — Additional file 1: Fig. S1. Wavelet amplitudes of neurogenic and endothelial NO-independent activities at phase 2 (prior to CIVD) in groups of subjects differentiated by their CIVD response and genotypes of (a) rs73619297 of COL4A2 or (b) rs931740 of PRLR. Data are means ± standard error of the mean. a.u., arbitrary units. Fig. S2. Principal component analysis of imputed genotype data from the JPQ cohort. Fig. S3. Wavelet amplitudes of endothelial NO-independent, endothelial NO-dependent, and neurogenic activities in four phases, including the baseline phase, in 10 study subjects with no CIVD response. Phases 1, 2, and 3 represent “vasoconstriction,” “prior to CIVD,” and “CIVD,” respectively [1]. a.u., arbitrary units. Fig. S4. Physical position and linkage disequilibrium of candidate SNPs around COL4A2 or at 14q23.3 in 94 Japanese subjects. The diagram was created using Haploview version 4.2. The numbers in diamonds represent r2 values (×100). The haplotype block was defined using the method of Gabriel et al. [2]. SNPs shown in bold are CIVD-associated SNPs. Table S1. Characteristics of all study subjects including one female. Table S2. SNP with p < 1×10^-5^ for CIVD-related categorical traits (CIVD response and eNO-independent activity, neurogenic activity) in the logistic regression analysis with the additive model. Table S3. SNP with p < 1×10^-5^ for CIVD-related categorical traits (CIVD response and eNO-independent activity, neurogenic activity) in the Fisher's exact test with the additive model. Table S4. Allele frequency of candidate SNPs detected in GWASs of CIVD response with neurogenic and endothelial NO-independent activities. Table S5. SNP with p < 1×10^-5^ for eNO-independent and neurogenic activities in the additive model using two-way ANOVA. Table S6. SNP with p < 1×10^-5^ for eNO-independent and neurogenic activities in the dominant model using two-way ANOVA. Table S7. Relationships among the genotypes of five candidate SNPs defined in the dominant model and [file 40101_2023_319_MOESM1_ESM.pdf]

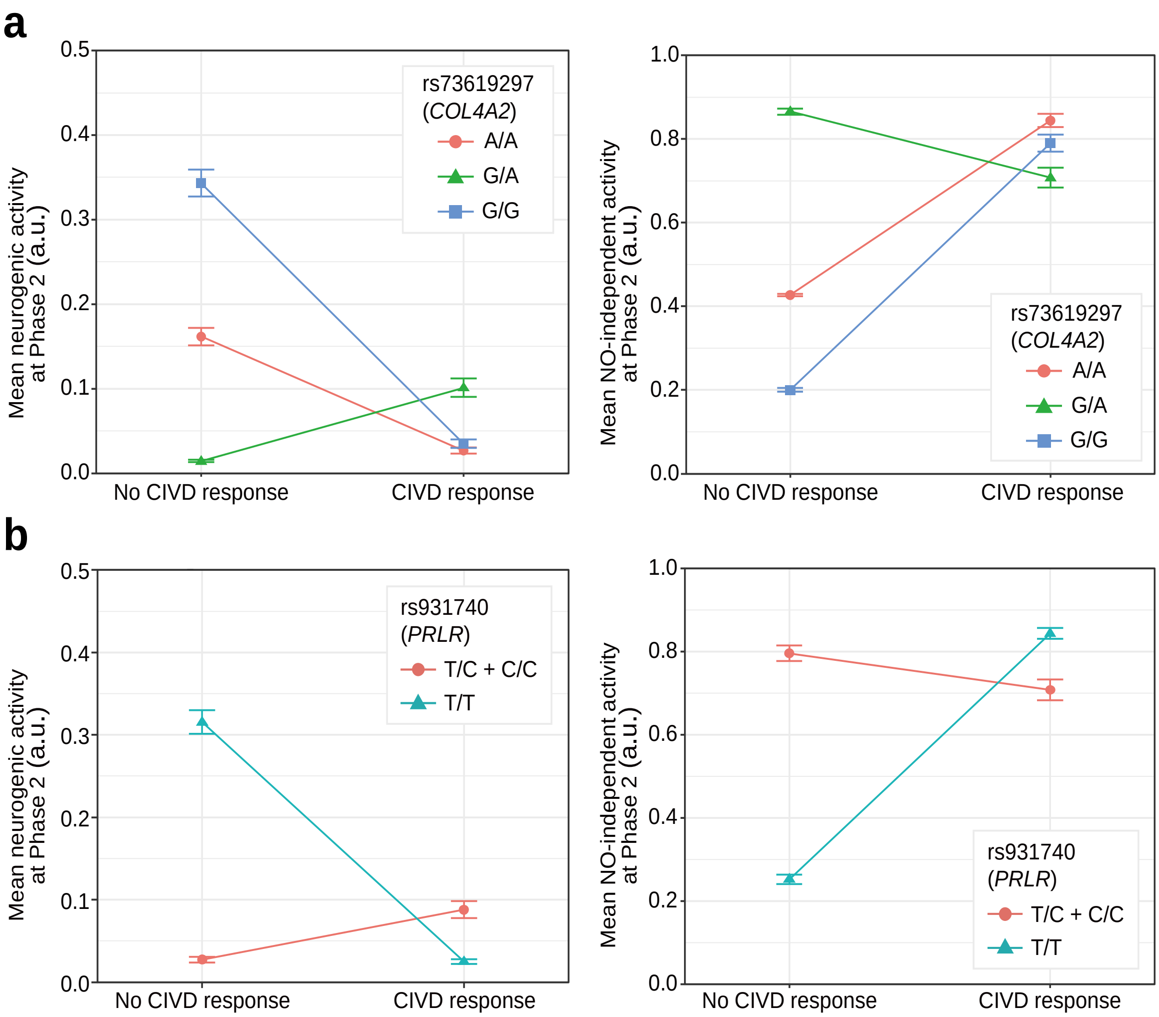

Fig. S1. Wavelet amplitudes of neurogenic and endothelial NO-independent activities at phase 2 (prior to CIVD) in groups of subjects differentiated by their CIVD response and genotypes of (a) rs73619297 of *COL4A2* or (b) rs931740 of *PRLR*. Data are means ± standard error of the mean. a.u., arbitrary units.

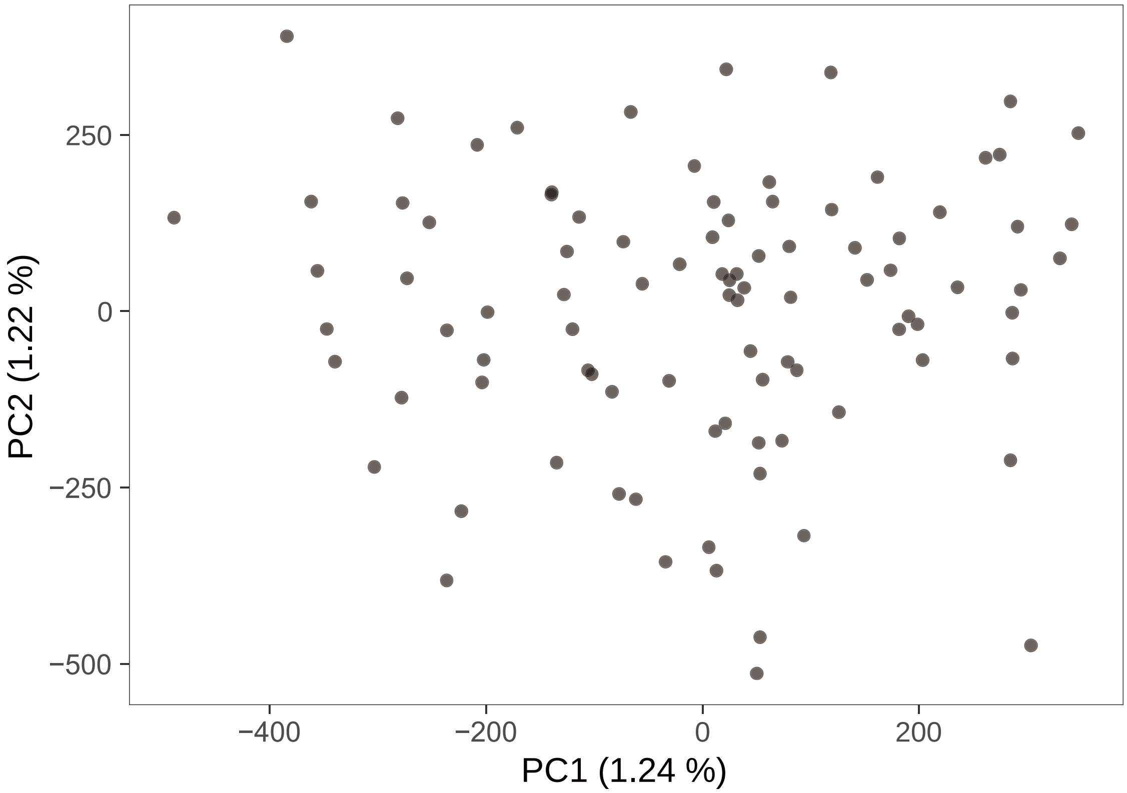

Fig. S2. Principal component analysis of imputed genotype data from the JPQ cohort.

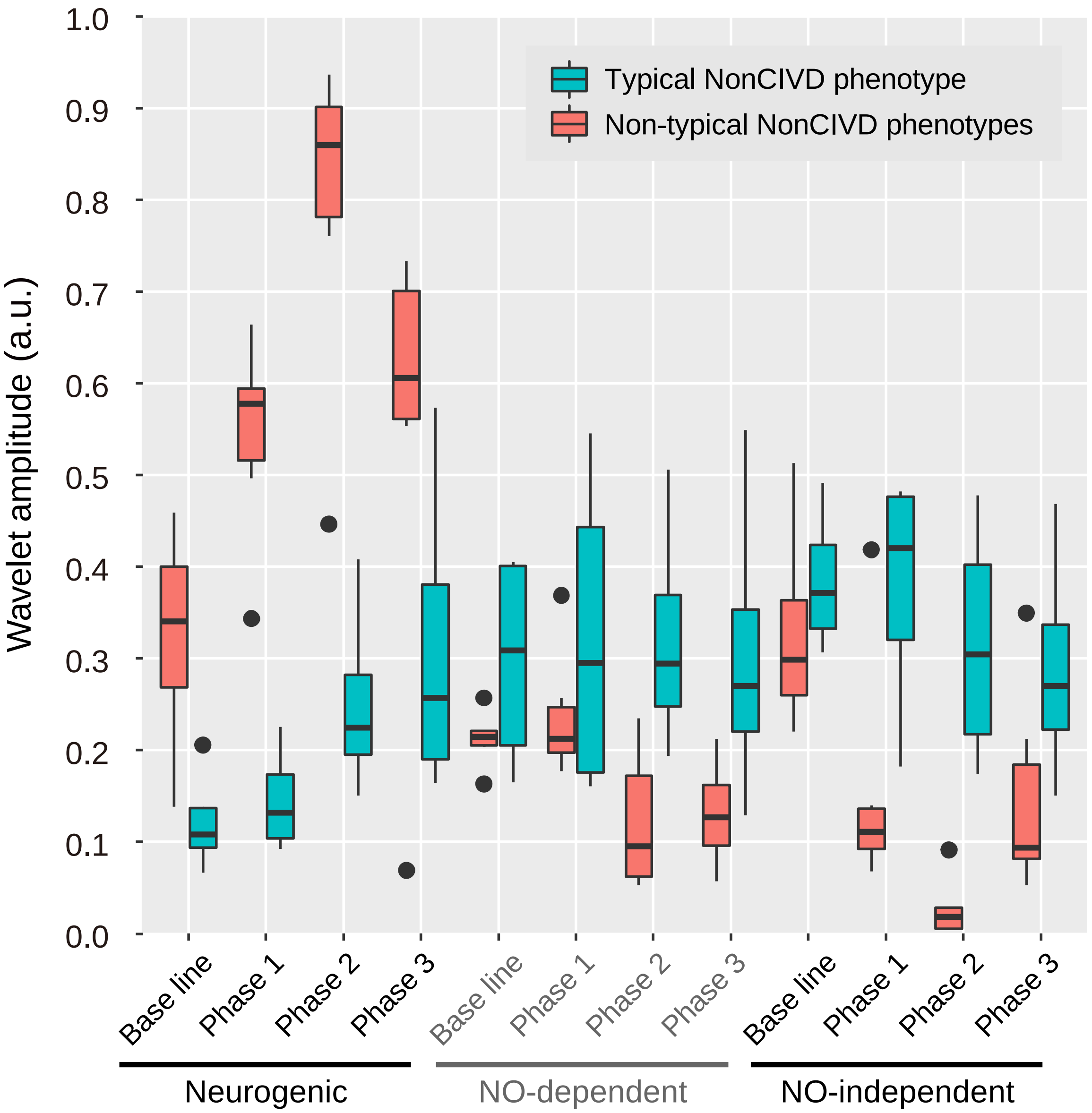

Fig. S3. Wavelet amplitudes of endothelial NO-independent, endothelial NO-dependent, and neurogenic activities in four phases, including the baseline phase, in 10 study subjects with no CIVD response. Phases 1, 2, and 3 represent “vasoconstriction,” “prior to CIVD,” and “CIVD,” respectively [1]. a.u., arbitrary units.

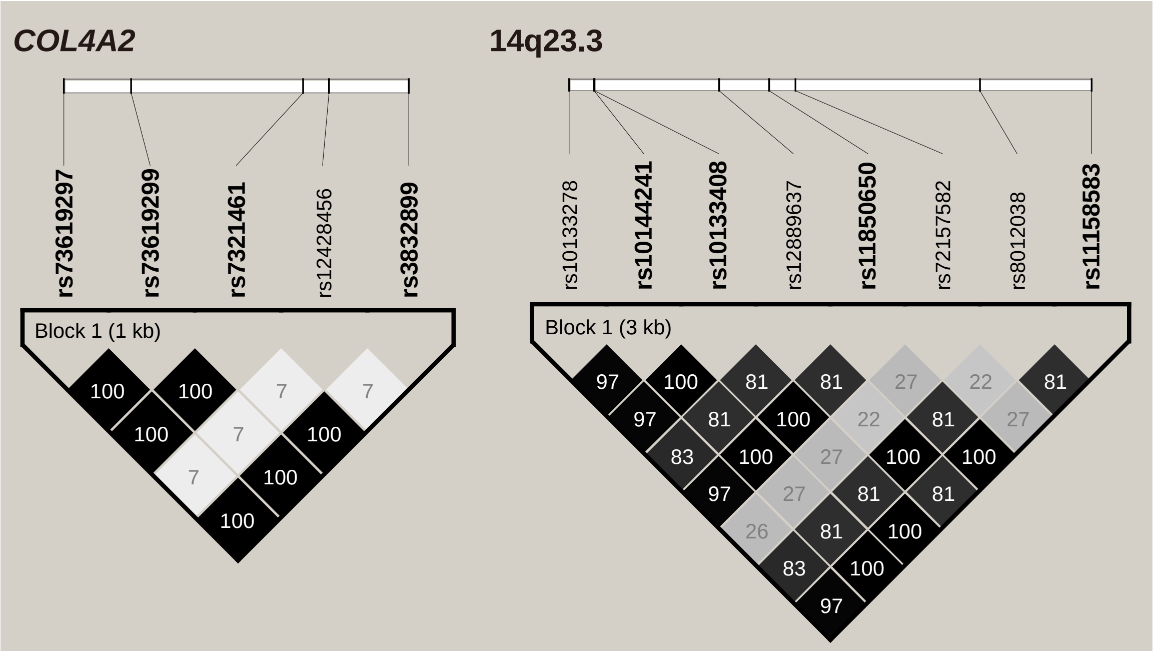

Fig. S4. Physical position and linkage disequilibrium of candidate SNPs around *COL4A2* or at 14q23.3 in 94 Japanese subjects. The diagram was created using Haploview version 4.2. The numbers in diamonds represent r^2 values (×100). The haplotype block was defined using the method of Gabriel et al. [2]. SNPs shown in bold are CIVD-associated SNPs.

Table S1. Characteristics of all study subjects including one female.

Characteristic	Mean ± SE	Minimum	Maximum	Female
Height, cm	171.78 ± 0.59	156.00	183.00	156.00
Weight, kg	64.03 ± 1.11	46.50	110.00	50.30
Body mass index, kg/m^2	21.68 ± 0.35	16.20	36.33	20.67
Body temperature, °C	36.46 ± 0.04	35.50	37.30	36.40
Neurogenic activity [a] (Baseline)	0.29 ± 0.01	0.03	0.56	0.38
Neurogenic activity [a] (Phase 1) [b]	0.47 ± 0.02	0.07	0.80	0.76
Neurogenic activity [a] (Phase 2) [b]	0.74 ± 0.02	0.15	0.99	0.96
Neurogenic activity [a] (Phase 3) [b]	0.57 ± 0.02	0.04	0.90	0.73
eNO-dependent activity [a] (Baseline)	0.25 ± 0.01	0.10	0.48	0.20
eNO-dependent activity [a] (Phase 1) [b]	0.26 ± 0.01	0.04	0.55	0.10
eNO-dependent activity [a] (Phase 2) [b]	0.15 ± 0.01	0.00	0.61	0.03
eNO-dependent activity [a] (Phase 3) [b]	0.17 ± 0.01	0.03	0.58	0.15
eNO-independent activity [a] (Baseline)	0.31 ± 0.01	0.17	0.51	0.26
eNO-independent activity [a] (Phase 1) [b]	0.20 ± 0.01	0.02	0.57	0.04
eNO-independent activity [a] (Phase 2) [b]	0.07 ± 0.01	0.00	0.48	0.01
eNO-independent activity [a] (Phase 3) [b]	0.15 ± 0.01	0.02	0.58	0.06

Abbreviations: eNO, endothelial nitric oxide; SE, standard error.
[a] Wavelet amplitude estimated from finger skin blood flow (arbitrary units).
[b] Phases 1, 2, and 3 represent “vasoconstriction,” “prior to CIVD,” and “CIVD,” respectively [1].

Table S2. SNP with $p < 1×10^{-5}$ for CIVD-related categorical traits (CIVD response and eNO-independent activity, neurogenic activity) in the logistic regression analysis with the additive model.

Trait	SNP	Chr	Position (GRCh37)	Empirical *p*-value
CIVD	rs80021844	3	166739035	2.32E-06
	rs10056404	5	76539997	3.04E-06
	rs276574	6	137434205	7.00E-06
	rs4895474	6	137434738	7.00E-06
	rs4895475	6	137435023	7.00E-06
	rs9483989	6	137435667	7.00E-06
	rs9373178	6	137436809	7.00E-06
	rs4896234	6	137437437	7.00E-06
	rs2327798	6	137437988	7.00E-06
	rs62420823	6	137438566	7.00E-06
	rs62420824	6	137438606	7.00E-06
	rs17252967	6	137438707	7.00E-06
	rs9389472	6	137439544	7.00E-06
	rs9494657	6	137439915	7.00E-06
	rs9402871	6	137440100	7.00E-06
	rs9402872	6	137440301	7.00E-06
	rs9399201	6	137441113	7.00E-06
	rs4896235	6	137441248	7.00E-06
	rs719640	6	137442746	7.00E-06

rs4895476	6	137443924	7.00E-06
rs9321587	6	137444356	7.00E-06
rs9373179	6	137444478	7.00E-06
rs9399202	6	137444523	7.00E-06
rs9385784	6	137445387	7.00E-06
rs3784333	15	78635188	7.04E-06
rs4886568	15	78637573	7.04E-06
rs16969725	15	78637938	7.04E-06
rs11686013	2	19344880	8.00E-06
rs1073009	2	19350640	8.00E-06
rs2083105	2	19353125	8.00E-06
rs2043748	2	19353635	8.00E-06
rs6705237	2	19355912	8.00E-06
rs6719583	2	19362446	8.00E-06
rs146079525	2	19362604	8.00E-06
rs6723401	2	19363418	8.00E-06
rs4569431	2	19363527	8.00E-06
rs10191271	2	19364202	8.00E-06
rs148846695	2	19365288	8.00E-06
rs9306880	2	19367112	8.00E-06
rs35122111	1	40472856	8.81E-06
rs35347867	1	40472864	8.81E-06
rs2118381	2	19368078	9.00E-06

	rs851340	2	19369285	9.00E-06
eNO-independent	rs167514	4	25234781	4.00E-06
	rs2654845	4	100370764	6.00E-06
	rs2851028	4	100357018	6.00E-06
	rs313556	4	25245446	4.00E-06
	rs371936876	4	25293618	8.00E-06
	rs4701735	5	6518007	5.32E-06
	rs9367155	6	42515315	8.00E-06
	rs9369370	6	42565741	5.00E-06
	rs6935425	6	5986521	7.00E-06
	rs10793846	6	5987845	7.00E-06
	rs143443868	6	42649322	6.00E-06
	rs17287873	6	42572859	5.00E-06
	rs1884086	6	42604320	5.00E-06
	rs2038261	6	42644623	8.00E-06
	rs2038640	6	5988749	5.00E-06
	rs2395922	6	42625386	5.00E-06
	rs2812674	6	42607056	5.00E-06
	rs4460209	6	42638564	8.00E-06
	rs4711725	6	42594053	5.00E-06
	rs55679749	6	42568082	5.00E-06
	rs55898675	6	42520396	8.00E-06
	rs57880987	6	42573152	5.00E-06

	rs6458288	6	42508329	8.00E-06
	rs6458299	6	42586058	5.00E-06
	rs6458300	6	42589262	5.00E-06
	rs6911657	6	42525949	5.00E-06
	rs6917033	6	42627434	4.00E-06
	rs7740672	6	42607331	5.00E-06
	rs7741465	6	42597065	5.00E-06
	rs7762500	6	42537205	5.00E-06
	rs9369374	6	42589838	5.00E-06
	rs9381189	6	42505139	8.00E-06
	rs9381190	6	42506402	8.00E-06
	rs9381191	6	42513111	8.00E-06
	rs9381196	6	42537803	7.00E-06
	rs35754014	8	90596618	9.00E-06
	rs10995858	10	66031698	3.11E-06
	rs11060001	12	129381753	3.00E-06
	rs11060002	12	129382245	4.01E-06
	rs1951192	14	32944107	2.00E-06
	rs112250269	14	32943585	2.00E-06
	rs745561	14	32944071	2.00E-06
	rs7170916	15	97992682	9.00E-06
Neurogenic	rs2395192	6	32447644	1.00E-06
	rs5020946	6	32450089	2.00E-06

rs17209866	6	32445992	2.00E-06
rs28895253	6	32444910	2.00E-06
rs28895254	6	32444946	2.00E-06
rs5020944	6	32450140	2.00E-06
rs5020947	6	32450033	2.00E-06
rs9394099	6	32449160	2.00E-06
rs747619	2	130305487	3.00E-06
rs10174479	2	130296287	3.00E-06
rs13002385	2	130276865	3.00E-06
rs1453147	2	130271510	3.00E-06
rs200743100	2	130292770	3.00E-06
rs28625431	2	130303333	3.00E-06
rs9268839	6	32428772	3.00E-06
rs12195582	6	32444544	3.00E-06
rs28895251	6	32444708	3.00E-06
NA	6	32444733	3.00E-06
rs9378212	6	32445691	3.00E-06
NA	6	32446425	3.00E-06
rs29001478	6	32447162	3.00E-06
rs29001568	6	32447341	3.00E-06
rs4410767	6	32448129	3.00E-06
rs9394098	6	32449138	3.00E-06
rs13194665	6	32451526	3.00E-06

rs13194770	6	32451570	3.00E-06
rs28561769	6	32452626	3.00E-06
rs12194148	6	32444198	3.00E-06
rs12195589	6	32444584	3.00E-06
rs12207473	6	32444330	3.00E-06
rs13211921	6	32442836	3.00E-06
rs138733099	6	32453101	3.00E-06
rs141688355	6	32452028	3.00E-06
rs17203514	6	32446071	3.00E-06
rs17203563	6	32446459	3.00E-06
rs17203619	6	32446922	3.00E-06
rs17203626	6	32446994	3.00E-06
rs17203636	6	32447014	3.00E-06
rs17209887	6	32446051	3.00E-06
rs1964995	6	32449411	3.00E-06
rs28662053	6	32453107	3.00E-06
rs28732244	6	32444096	3.00E-06
rs28732246	6	32447219	3.00E-06
rs28732247	6	32447474	3.00E-06
rs28893500	6	32444511	3.00E-06
rs28895242	6	32443666	3.00E-06
rs28895247	6	32443890	3.00E-06
rs28895249	6	32444021	3.00E-06

rs28895255	6	32445079	3.00E-06
rs28895257	6	32445114	3.00E-06
rs28895258	6	32445117	3.00E-06
rs28895259	6	32445258	3.00E-06
rs28895261	6	32445306	3.00E-06
rs28895265	6	32445793	3.00E-06
rs28895267	6	32445916	3.00E-06
rs29001620	6	32447216	3.00E-06
rs29001652	6	32447111	3.00E-06
rs34207893	6	32447616	3.00E-06
rs34452456	6	32450678	3.00E-06
rs35007143	6	32447518	3.00E-06
rs35875942	6	32447563	3.00E-06
rs4457186	6	32447653	3.00E-06
rs4994854	6	32447900	3.00E-06
rs4994855	6	32447873	3.00E-06
rs4994859	6	32447715	3.00E-06
rs5018660	6	32449293	3.00E-06
rs62405566	6	32453095	3.00E-06
rs71543453	6	32452361	3.00E-06
rs71543454	6	32452399	3.00E-06
rs71543455	6	32452409	3.00E-06
rs7452937	6	32446484	3.00E-06

rs75259234	6	32453338	3.00E-06
rs77469652	6	32453532	3.00E-06
rs9378264	6	32443451	3.00E-06
rs9391879	6	32443172	3.00E-06
rs9391881	6	32443432	3.00E-06
rs28895252	6	32444709	4.00E-06
rs115610745	6	32453632	4.00E-06
rs17203612	6	32446853	4.00E-06
rs28895241	6	32443665	4.00E-06
rs9378266	6	32448189	4.00E-06
rs4711298	6	32443804	4.02E-06
rs17209873	6	32446010	5.00E-06
rs2406981	2	75363423	5.04E-06
rs56194380	11	99598563	5.81E-06
NA	6	32452602	6.00E-06
rs9469126	6	32443866	6.02E-06
rs741418	2	75363186	6.04E-06
rs3797288	5	53212749	7.00E-06
rs3130267	6	33306794	8.00E-06
rs3130262	6	33300854	8.00E-06
rs3130264	6	33301229	8.00E-06
rs3130265	6	33302886	8.00E-06
rs34614226	6	32554963	8.00E-06

rs144150162	6	32457348	9.00E-06
rs34303755	6	32450613	9.00E-06
rs79948287	3	3272111	9.00E-06
rs117330953	3	3271699	9.00E-06
rs12632210	3	3274803	9.00E-06
rs61048867	3	3273331	9.00E-06
rs76688955	3	3273449	9.00E-06

Abbreviations: Chr, chromosome; CIVD, cold-induced vasodilation; eNO, endothelial nitric oxide; NA, not applicable.

Table S3. SNP with $p < 1×10^{-5}$ for CIVD-related categorical traits (CIVD response and eNO-independent activity, neurogenic activity) in the Fisher's exact test with the additive model.

Trait	SNP	Chr	Position (GRCh37)	Empirical *p*-value
CIVD	rs2297495	1	62457365	3.00.E-06
	rs3762445	1	62455658	3.00.E-06
	rs10493315	1	62459477	3.00.E-06
	rs10493316	1	62458463	3.00.E-06
	rs10493317	1	62457712	3.00.E-06
	rs17122892	1	62454784	3.00.E-06
	rs67417917	1	62460764	3.00.E-06
	rs67719504	1	62460034	3.00.E-06
	rs67967624	1	62459970	3.00.E-06
	rs7538799	1	62456869	4.00.E-06
	rs7668968	4	66726174	4.00.E-06
	rs2350077	4	66721398	4.00.E-06
	rs2760473	1	59306057	5.00.E-06
	rs1073009	2	19350640	5.50.E-06
	rs2043748	2	19353635	5.50.E-06
	rs2083105	2	19353125	5.50.E-06
	rs4569431	2	19363527	5.50.E-06
	rs6705237	2	19355912	5.50.E-06
	rs6719583	2	19362446	5.50.E-06
	rs6723401	2	19363418	5.50.E-06

rs9306880	2	19367112	5.50.E-06
rs10191271	2	19364202	5.50.E-06
rs11686013	2	19344880	5.50.E-06
rs146079525	2	19362604	5.50.E-06
rs148846695	2	19365288	5.50.E-06
rs2118381	2	19368078	6.00.E-06
rs851340	2	19369285	6.00.E-06
rs12710701	2	19344757	6.00.E-06
rs80021844	3	166739035	7.00.E-06
rs17122915	1	62477947	8.00.E-06
rs17122904	1	62472142	8.00.E-06
rs67035896	1	62471244	8.00.E-06
rs67526244	1	62469455	8.00.E-06
rs67622686	1	62475063	8.00.E-06
rs67867258	1	62475973	8.00.E-06
rs113302608	1	62465912	8.00.E-06
rs201386776	1	62463918	8.00.E-06
rs142990923	1	62464281	8.00.E-06
rs12471909	2	19349554	8.00.E-06
rs12474324	2	19346130	8.00.E-06
rs12476121	2	19352500	8.00.E-06
rs55895241	2	19361681	8.00.E-06
rs55960635	2	19349068	8.00.E-06

	rs75216565	2	19367568	8.00.E-06
	rs75454728	2	19356862	8.00.E-06
	rs76564774	2	19368696	8.00.E-06
	rs80348365	2	19347703	8.00.E-06
	rs370304002	2	19365846	8.00.E-06
eNO-independent	rs74922259	2	19342228	1.50.E-06
	rs4666462	2	19317004	1.50.E-06
	rs6761692	2	19339938	1.50.E-06
	rs12464722	2	19341657	1.50.E-06
	rs12471909	2	19349554	1.50.E-06
	rs12474324	2	19346130	1.50.E-06
	rs12476121	2	19352500	1.50.E-06
	rs55895241	2	19361681	1.50.E-06
	rs55960635	2	19349068	1.50.E-06
	rs74328396	2	19330626	1.50.E-06
	rs75216565	2	19367568	1.50.E-06
	rs75454728	2	19356862	1.50.E-06
	rs76029180	2	19324263	1.50.E-06
	rs76384991	2	19309906	1.50.E-06
	rs76564774	2	19368696	1.50.E-06
	rs78085478	2	19330230	1.50.E-06
	rs79584745	2	19326239	1.50.E-06
	rs79845868	2	19312190	1.50.E-06

rs79912139	2	19328087	1.50.E-06
rs80348365	2	19347703	1.50.E-06
rs370304002	2	19365846	1.50.E-06
rs140023049	2	19314293	1.50.E-06
rs140791157	2	19334085	2.00.E-06
rs74041632	14	33013657	5.50.E-06
rs78366435	14	33012556	5.50.E-06
rs7958507	12	22250653	8.00.E-06
rs2160629	12	22250528	8.00.E-06
rs7134810	12	22251266	8.00.E-06
rs7304188	12	22251217	8.00.E-06
rs7304301	12	22251264	8.00.E-06
rs7312934	12	22253333	8.00.E-06
rs7958351	12	22250542	8.00.E-06
rs11536005	12	22253468	8.00.E-06

Abbreviations: Chr, chromosome; CIVD, cold-induced vasodilation; eNO, endothelial nitric oxide.
No genetic variants with $p < 1\times10^{-5}$ were detected in the Fisher's exact test for neurogenic activity.

Table S4. Allele frequency of candidate SNPs detected in GWASs of CIVD response with neurogenic and endothelial NO-independent activities.

RefSNP ID	Position[a]	Gene	Allele frequency[b]		H_{obs}	H_{exp}	HWE (*P*-value)[c]
			Major	Minor			
rs17113836	5: 152344712	*LINC01470*	A: 0.72 (134)	T: 0.28 (52)	0.452	0.403	0.310
rs73619297	13: 110967247	*COL4A2*	G: 0.62 (116)	A: 0.38 (72)	0.447	0.473	0.662
rs73619299	13: 110967456	*COL4A2*	C: 0.62 (116)	T: 0.38 (72)	0.447	0.473	0.662
rs7321461	13: 110967990	*COL4A2*	A: 0.62 (116)	C: 0.38 (72)	0.447	0.473	0.662
rs3832899	13: 110968318	*COL4A2*	T: 0.62 (116)	–: 0.38 (72)	0.447	0.473	0.662
rs931740	5: 35222040	*PRLR*	T: 0.59 (109)	C: 0.41 (75)	0.424	0.483	0.280
rs10144241	14: 65638439	14q23.3	C: 0.57 (108)	A: 0.43 (80)	0.447	0.489	0.404
rs10133408	14: 65638443	14q23.3	T: 0.57 (108)	G: 0.43 (80)	0.447	0.489	0.404
rs11850650	14: 65639582	14q23.3	T: 0.57 (108)	C: 0.43 (80)	0.447	0.489	0.404
rs11158583	14: 65641686	14q23.3	G: 0.57 (108)	C: 0.43 (80)	0.447	0.489	0.404

Abbreviations: CIVD, cold-induced vasodilation; NO, nitric oxide; SNP, single-nucleotide polymorphism; H_{obs}, observed heterozygosity; H_{exp}, expected heterozygosity; HWE, Hardy–Weinberg equilibrium.

[a] Chromosomal position in NCBI build GRCh37.

[b] Values indicate allele frequencies with the sample numbers indicated in parentheses.

[c] Probability of a genetic variant for which the genotype distribution does not deviate from the HWE (according to Fisher's exact test).

Table S5. SNP with $p < 1\times10^{-5}$ for eNO-independent and neurogenic activities in the additive model using two-way ANOVA.

SNP	Chr	Position (GRCh37)	Position (GRCh38)	*p* (eNO-independent)	*p* (neurogenic)
rs17113836	5	152344712	152965152	7.02E-07	1.89E-08
rs73619297	13	110967247	110314900	9.36E-06	3.24E-08
rs73619299	13	110967456	110315109	9.36E-06	3.24E-08
rs7321461	13	110967990	110315643	9.36E-06	3.24E-08
rs3832899	13	110968318	110315971	9.36E-06	3.24E-08
rs11586897	1	112501173	111958551	8.28E-06	5.08E-08
rs1373290	1	112501812	111959190	8.28E-06	5.08E-08
rs11902653	2	167997478	167140968	9.77E-06	6.52E-08
rs73017939	2	168004354	167147844	9.77E-06	6.52E-08
rs6740526	2	168006149	167149639	9.77E-06	6.52E-08
rs11888230	2	168007034	167150524	9.77E-06	6.52E-08
rs4502379	2	168010163	167153653	9.77E-06	6.52E-08
rs113155011	2	168020722	167164212	9.77E-06	6.52E-08
rs11893404	2	168020995	167164485	9.77E-06	6.52E-08
rs11893377	2	168021121	167164611	9.77E-06	6.52E-08
rs11893509	2	168021232	167164722	9.77E-06	6.52E-08
rs7556854	2	168021731	167165221	9.77E-06	6.52E-08
rs67386767	2	168024025	167167515	9.77E-06	6.52E-08
rs55733637	2	168029549	167173039	9.77E-06	6.52E-08
rs58616240	2	168029842	167173332	9.77E-06	6.52E-08

rs80110888	5	152348048	152968488	2.38E-06	1.26E-07
rs60328726	5	169832592	170405588	7.38E-06	1.46E-07
rs143434516	5	169833188	170406184	7.38E-06	1.46E-07
rs75661496	5	169833745	170406741	7.38E-06	1.46E-07
rs7722552	5	169834501	170407497	7.38E-06	1.46E-07
rs10051800	5	169835138	170408134	7.38E-06	1.46E-07
rs17740022	5	169835524	170408520	7.74E-06	1.58E-07
rs77273451	5	169835858	170408854	9.85E-06	2.01E-07
rs2656848	5	169837712	170410708	9.85E-06	2.01E-07
rs58322156	4	15828382	15826759	8.84E-06	3.38E-07
rs7159808	14	55624327	55157609	1.30E-06	3.55E-07
rs4331145	11	643683	643683	5.26E-06	4.03E-07
rs200635951	12	97277789	96884011	2.56E-06	4.42E-07
rs4820715	22	27287669	26891706	3.60E-06	5.27E-07
rs6819850	4	4784097	4782370	2.41E-06	5.87E-07
rs55844046	12	97234396	96840618	1.43E-06	6.04E-07
rs28740902	4	4792560	4790833	1.52E-06	9.02E-07
rs7663600	4	4786727	4785000	2.39E-06	1.08E-06
rs7469786	9	82558699	79943784	7.99E-06	1.11E-06
rs10867448	9	82565958	79951043	7.99E-06	1.11E-06
rs71498102	9	86994548	84379633	8.43E-06	1.20E-06
rs5761811	22	27285154	26889191	5.34E-06	1.25E-06
rs5761812	22	27285261	26889298	5.34E-06	1.25E-06

rs5761813	22	27285813	26889850	6.06E-06	1.46E-06
rs5761817	22	27286859	26890896	6.06E-06	1.46E-06
rs5752434	22	27286864	26890901	6.06E-06	1.46E-06
rs5761818	22	27287053	26891090	6.06E-06	1.46E-06
rs4822812	22	27287869	26891906	6.06E-06	1.46E-06
rs11090459	22	27287946	26891983	6.06E-06	1.46E-06
rs13057132	22	27288005	26892042	6.06E-06	1.46E-06
rs5752436	22	27288251	26892288	6.06E-06	1.46E-06
rs5752437	22	27288263	26892300	6.06E-06	1.46E-06
rs5761820	22	27288275	26892312	6.06E-06	1.46E-06
rs35900285	12	97188218	96794440	2.94E-06	1.60E-06
rs79419580	4	83317024	82395871	1.05E-06	1.61E-06
rs441399	15	91366222	90822992	2.69E-07	1.63E-06
rs6817807	4	4776519	4774792	4.55E-06	1.77E-06
rs9291151	4	4775137	4773410	5.11E-06	1.82E-06
rs6817123	4	4776774	4775047	5.11E-06	1.82E-06
rs7298635	12	97166675	96772897	3.88E-06	1.89E-06
rs5800270	12	97166795	96773017	3.88E-06	1.89E-06
rs5800271	12	97167028	96773250	3.88E-06	1.89E-06
rs10777815	12	97167292	96773514	3.88E-06	1.89E-06
rs6538740	12	97168032	96774254	3.88E-06	1.89E-06
rs7133416	12	97168950	96775172	3.88E-06	1.89E-06
rs4254116	12	97170447	96776669	3.88E-06	1.89E-06

rs7962554	12	97171218	96777440	3.88E-06	1.89E-06
rs7962868	12	97171319	96777541	3.88E-06	1.89E-06
rs11108693	12	97172138	96778360	3.88E-06	1.89E-06
rs7309462	12	97179688	96785910	3.88E-06	1.89E-06
rs11108696	12	97180430	96786652	3.88E-06	1.89E-06
rs10610138	12	97181107	96787329	3.88E-06	1.89E-06
rs11366923	12	97183790	96790012	3.88E-06	1.89E-06
rs7315634	12	97184036	96790258	3.88E-06	1.89E-06
rs141266071	12	97184436	96790658	3.88E-06	1.89E-06
rs11108697	12	97185646	96791868	3.88E-06	1.89E-06
rs1833243	12	97187722	96793944	3.88E-06	1.89E-06
rs12227033	12	97190477	96796699	3.88E-06	1.89E-06
rs11108698	12	97191072	96797294	3.88E-06	1.89E-06
rs7307920	12	97191681	96797903	3.88E-06	1.89E-06
rs7301455	12	97202567	96808789	3.88E-06	1.89E-06
rs7301971	12	97202700	96808922	3.88E-06	1.89E-06
rs11108702	12	97203440	96809662	3.88E-06	1.89E-06
rs6538743	12	97205338	96811560	3.88E-06	1.89E-06
rs11108704	12	97209077	96815299	3.88E-06	1.89E-06
rs68085226	12	97211401	96817623	3.88E-06	1.89E-06
rs147421916	12	97211403	96817625	3.88E-06	1.89E-06
rs386765541	12	97211405	96817627	3.88E-06	1.89E-06
rs149918289	12	97211407	96817629	3.88E-06	1.89E-06

rs142077887	12	97211410	96817632	3.88E-06	1.89E-06
rs970620	12	97211528	96817750	3.88E-06	1.89E-06
rs2041913	12	97216465	96822687	3.88E-06	1.89E-06
No_rs_ID	12	97220312	96826534	3.88E-06	1.89E-06
rs11108713	12	97224280	96830502	3.88E-06	1.89E-06
rs12316471	12	97230573	96836795	3.88E-06	1.89E-06
rs7136658	12	97231171	96837393	3.88E-06	1.89E-06
rs72214158	12	97234395	96840617	3.88E-06	1.89E-06
rs11108716	12	97234942	96841164	3.88E-06	1.89E-06
rs2014710	12	97238307	96844529	3.88E-06	1.89E-06
rs10860100	12	97240587	96846809	3.88E-06	1.89E-06
rs6538744	12	97245096	96851318	3.88E-06	1.89E-06
rs2371380	12	97250453	96856675	3.88E-06	1.89E-06
rs1976902	2	9787131	9647002	6.48E-06	1.95E-06
rs1035203	12	97226589	96832811	3.95E-06	2.04E-06
rs28827865	4	167567976	166646825	2.74E-06	2.21E-06
rs11736191	4	4768475	4766748	5.19E-06	2.47E-06
rs7687367	4	4770225	4768498	5.19E-06	2.47E-06
rs6432027	2	9784741	9644612	9.25E-06	2.50E-06
rs73913150	2	9785267	9645138	8.30E-06	2.56E-06
rs73913152	2	9785377	9645248	8.30E-06	2.56E-06
rs10495569	2	9784356	9644227	8.62E-06	2.56E-06
rs2691258	19	51517286	51014030	5.57E-06	3.08E-06

rs2075691	19	51519114	51015858	5.57E-06	3.08E-06
rs2075690	19	51519236	51015980	5.57E-06	3.08E-06
rs71460363	12	97270915	96877137	6.70E-06	3.11E-06
rs7296042	12	97271555	96877777	6.70E-06	3.11E-06
rs11108724	12	97274404	96880626	6.70E-06	3.11E-06
rs7956695	12	97277027	96883249	6.70E-06	3.11E-06
rs2213771	22	27271926	26875963	9.95E-07	3.32E-06
rs13118887	4	138328077	137406923	8.22E-06	4.00E-06
rs11732448	4	138328340	137407186	8.22E-06	4.00E-06
rs4864419	4	138329927	137408773	8.22E-06	4.00E-06
rs12643561	4	138330598	137409444	8.22E-06	4.00E-06
rs17049558	4	138331082	137409928	8.22E-06	4.00E-06
rs10008144	4	138331602	137410448	8.22E-06	4.00E-06
rs28639939	4	138332718	137411564	8.22E-06	4.00E-06
rs2406733	4	138333845	137412691	8.22E-06	4.00E-06
rs10967689	9	27066281	27066283	5.11E-06	4.07E-06
rs10854283	21	15497145	14124824	4.44E-06	4.29E-06
rs2454986	10	57906113	56146352	1.00E-06	4.51E-06
rs7100770	10	129477336	127679072	2.70E-06	4.52E-06
rs6665337	1	58849912	58384240	4.20E-06	4.88E-06
rs55875707	1	58850101	58384429	4.20E-06	4.88E-06
rs4145691	10	57931055	56171294	8.87E-07	4.90E-06
rs7916378	10	57931491	56171730	8.87E-07	4.90E-06

rs62208642	21	15489399	14117078	6.77E-06	5.32E-06
rs2403729	21	15491560	14119239	6.77E-06	5.32E-06
rs139972366	21	15493142	14120821	6.77E-06	5.32E-06
rs2032283	21	15494955	14122634	6.77E-06	5.32E-06
rs2096926	21	15503493	14131172	6.77E-06	5.32E-06
rs12627207	21	15497019	14124698	6.74E-06	5.49E-06
rs2632723	11	73713129	74002084	9.52E-06	5.51E-06
rs1603785	15	27940159	27695013	1.10E-06	5.66E-06
rs2586796	17	71872022	73875883	5.65E-06	6.19E-06
rs62209377	21	15508891	14136570	6.81E-06	6.20E-06
rs9893718	17	30001835	31674816	9.61E-06	6.48E-06
rs2585348	14	49125132	48655929	9.68E-06	6.53E-06
rs1953894	14	49126265	48657062	9.68E-06	6.53E-06
rs199716451	14	49127972	48658769	9.68E-06	6.53E-06
rs7153673	14	49128088	48658885	9.68E-06	6.53E-06
rs11846647	14	49128128	48658925	9.68E-06	6.53E-06
rs12898134	14	49129400	48660197	9.68E-06	6.53E-06
rs12323405	14	49132021	48662818	9.68E-06	6.53E-06
rs2790559	14	49132280	48663077	9.68E-06	6.53E-06
rs7020413	9	37002115	37002118	1.55E-06	6.57E-06
rs1553276	4	16569992	16568369	4.62E-06	6.59E-06
rs10967631	9	26968344	26968346	6.11E-06	6.72E-06
rs1553275	4	16569922	16568299	5.60E-06	7.72E-06

rs388683	21	28234072	26861753	8.75E-06	8.02E-06
rs452359	21	28234078	26861759	8.75E-06	8.02E-06
rs397194	21	28234082	26861763	8.75E-06	8.02E-06
rs423048	21	28234284	26861965	8.75E-06	8.02E-06
rs410349	21	28235194	26862875	8.75E-06	8.02E-06
rs2016800	21	28235755	26863436	8.75E-06	8.02E-06
rs229104	21	28236050	26863731	8.75E-06	8.02E-06
rs229105	21	28237311	26864992	8.75E-06	8.02E-06
rs229106	21	28237348	26865029	8.75E-06	8.02E-06
rs229107	21	28237747	26865428	8.75E-06	8.02E-06
rs229108	21	28239997	26867678	8.75E-06	8.02E-06
rs35290138	21	28240014	26867695	8.75E-06	8.02E-06
rs3085100	21	28241111	26868792	8.75E-06	8.02E-06
rs229111	21	28241600	26869281	8.75E-06	8.02E-06
rs229112	21	28242858	26870539	8.75E-06	8.02E-06
rs2174222	4	18160108	18158485	9.43E-06	8.18E-06
rs6497227	15	27940585	27695439	3.16E-06	8.57E-06
rs6497228	15	27940854	27695708	3.16E-06	8.57E-06
rs9280179	6	32682918	32715141	6.82E-06	8.60E-06
rs4265062	6	32745886	32778109	6.82E-06	8.60E-06
rs7760168	6	32746051	32778274	6.82E-06	8.60E-06
rs7760779	6	32746464	32778687	6.82E-06	8.60E-06
rs4563763	6	32750292	32782515	6.82E-06	8.60E-06

rs7749543	6	32751416	32783639	6.82E-06	8.60E-06
rs9276708	6	32756917	32789140	6.82E-06	8.60E-06
rs403084	5	148819714	149440151	9.71E-06	8.76E-06
rs2331308	22	27261517	26865554	3.19E-06	9.14E-06
rs2331309	22	27261577	26865614	3.19E-06	9.14E-06
rs34403973	22	27261763	26865800	3.19E-06	9.14E-06
rs2331310	22	27261845	26865882	3.19E-06	9.14E-06
rs2105933	22	27262569	26866606	3.19E-06	9.14E-06
rs9608527	22	27263046	26867083	3.19E-06	9.14E-06
rs5761798	22	27265239	26869276	3.19E-06	9.14E-06
rs5761800	22	27266472	26870509	3.19E-06	9.14E-06
rs6005216	22	27267344	26871381	3.19E-06	9.14E-06
rs5761801	22	27268143	26872180	3.19E-06	9.14E-06
rs2157467	22	27269938	26873975	3.19E-06	9.14E-06
rs2157468	22	27270068	26874105	3.19E-06	9.14E-06
rs5761803	22	27272399	26876436	3.19E-06	9.14E-06
rs4778170	15	27941897	27696751	3.34E-06	9.49E-06

Chr, chromosome; eNO, endothelial nitric oxide; SNP, single-nucleotide polymorphism.

Table S6. SNP with $p < 1\times10^{-5}$ for eNO-independent and neurogenic activities in the dominant model using two-way ANOVA.

SNP	Chr	Position (GRCh37)	Position (GRCh38)	*p* (eNO-independent)	*p* (neurogenic)
rs931740	5	35222040	35221938	3.29E-06	1.03E-08
rs10144241	14	65638439	65171721	9.39E-06	4.78E-08
rs10133408	14	65638443	65171725	9.39E-06	4.78E-08
rs11850650	14	65639582	65172864	9.39E-06	4.78E-08
rs11158583	14	65641686	65174968	9.39E-06	4.78E-08
rs17113836	5	152344712	152965152	1.94E-06	5.34E-08
rs11902653	2	167997478	167140968	7.75E-06	5.16E-08
rs73017939	2	168004354	167147844	7.75E-06	5.16E-08
rs6740526	2	168006149	167149639	7.75E-06	5.16E-08
rs11888230	2	168007034	167150524	7.75E-06	5.16E-08
rs4502379	2	168010163	167153653	7.75E-06	5.16E-08
rs113155011	2	168020722	167164212	7.75E-06	5.16E-08
rs11893404	2	168020995	167164485	7.75E-06	5.16E-08
rs11893377	2	168021121	167164611	7.75E-06	5.16E-08
rs11893509	2	168021232	167164722	7.75E-06	5.16E-08
rs7556854	2	168021731	167165221	7.75E-06	5.16E-08
rs67386767	2	168024025	167167515	7.75E-06	5.16E-08
rs55733637	2	168029549	167173039	7.75E-06	5.16E-08
rs58616240	2	168029842	167173332	7.75E-06	5.16E-08
rs80110888	5	152348048	152968488	9.55E-06	5.36E-08

Chr, chromosome; eNO, endothelial nitric oxide; SNP, single-nucleotide polymorphism.

Table S7. Relationships among the genotypes of five candidate SNPs defined in the dominant model and network of three CIVD-related factors.

			Significant SNPs in the dominant model				
CIVD response	Neuro	eNO-indep	rs931740	rs10144241	rs10133408	rs11850650	rs11158583
Absence	Low	High	T/C	A/A	G/G	C/C	C/C
Absence	Low	High	T/C	A/A	G/G	C/C	C/C
Absence	Low	High	T/C	A/A	G/G	C/C	C/C
Absence	Low	High	T/C	C/A	T/G	T/C	G/C
Absence	Low	High	C/C	C/A	T/G	T/C	G/C
Absence	Low	Low	C/C	C/A	T/G	T/C	G/C
Absence	High	Low	T/T	C/C	T/T	T/T	G/G
Absence	High	Low	T/T	C/C	T/T	T/T	G/G
Absence	High	Low	T/T	C/C	T/T	T/T	G/G
Absence	High	Low	T/T	C/C	T/T	T/T	G/G
Presence	Low	High	C/C	A/A	G/G	C/C	C/C
Presence	Low	High	C/C	A/A	G/G	C/C	C/C
Presence	Low	High	NA	A/A	G/G	C/C	C/C
Presence	Low	High	T/C	A/A	G/G	C/C	C/C
Presence	Low	High	T/C	A/A	G/G	C/C	C/C
Presence	Low	High	T/C	A/A	G/G	C/C	C/C
Presence	Low	High	T/C	A/A	G/G	C/C	C/C
Presence	Low	High	T/C	A/A	G/G	C/C	C/C
Presence	Low	High	T/C	A/A	G/G	C/C	C/C
Presence	Low	High	T/T	A/A	G/G	C/C	C/C
Presence	Low	High	T/T	A/A	G/G	C/C	C/C
Presence	Low	High	T/T	A/A	G/G	C/C	C/C
Presence	Low	High	T/T	A/A	G/G	C/C	C/C
Presence	Low	High	C/C	C/A	T/G	T/C	G/C
Presence	Low	High	C/C	C/A	T/G	T/C	G/C
Presence	Low	High	C/C	C/A	T/G	T/C	G/C
Presence	Low	High	C/C	C/A	T/G	T/C	G/C
Presence	Low	High	NA	C/A	T/G	T/C	G/C
Presence	Low	High	T/C	C/A	T/G	T/C	G/C
Presence	Low	High	T/C	C/A	T/G	T/C	G/C
Presence	Low	High	T/C	C/A	T/G	T/C	G/C
Presence	Low	High	T/C	C/A	T/G	T/C	G/C
Presence	Low	High	T/C	C/A	T/G	T/C	G/C
Presence	Low	High	T/C	C/A	T/G	T/C	G/C
Presence	Low	High	T/C	C/A	T/G	T/C	G/C
Presence	Low	High	T/T	C/A	T/G	T/C	G/C
Presence	Low	High	T/T	C/A	T/G	T/C	G/C
Presence	Low	High	T/T	C/A	T/G	T/C	G/C
Presence	Low	High	T/T	C/A	T/G	T/C	G/C
Presence	Low	High	T/T	C/A	T/G	T/C	G/C
Presence	Low	High	T/T	C/A	T/G	T/C	G/C
Presence	Low	High	T/T	C/A	T/G	T/C	G/C
Presence	Low	High	T/T	C/A	T/G	T/C	G/C
Presence	Low	High	T/T	C/A	T/G	T/C	G/C
Presence	Low	High	T/T	C/A	T/G	T/C	G/C
Presence	Low	High	T/T	C/A	T/G	T/C	G/C
Presence	Low	High	T/T	C/A	T/G	T/C	G/C
Presence	Low	High	T/T	C/A	T/G	T/C	G/C
Presence	Low	High	T/T	C/A	T/G	T/C	G/C
Presence	Low	High	C/C	C/C	T/T	T/T	G/G
Presence	Low	High	C/C	C/C	T/T	T/T	G/G

Presence	Low	High	C/C	C/C	T/T	T/T	G/G
Presence	Low	High	C/C	C/C	T/T	T/T	G/G
Presence	Low	High	C/C	C/C	T/T	T/T	G/G
Presence	Low	High	C/C	C/C	T/T	T/T	G/G
Presence	Low	High	T/C	C/C	T/T	T/T	G/G
Presence	Low	High	T/C	C/C	T/T	T/T	G/G
Presence	Low	High	T/C	C/C	T/T	T/T	G/G
Presence	Low	High	T/C	C/C	T/T	T/T	G/G
Presence	Low	High	T/C	C/C	T/T	T/T	G/G
Presence	Low	High	T/C	C/C	T/T	T/T	G/G
Presence	Low	High	T/C	C/C	T/T	T/T	G/G
Presence	Low	High	T/C	C/C	T/T	T/T	G/G
Presence	Low	High	T/C	C/C	T/T	T/T	G/G
Presence	Low	High	T/C	C/C	T/T	T/T	G/G
Presence	Low	High	T/T	C/C	T/T	T/T	G/G
Presence	Low	High	T/T	C/C	T/T	T/T	G/G
Presence	Low	High	T/T	C/C	T/T	T/T	G/G
Presence	Low	High	T/T	C/C	T/T	T/T	G/G
Presence	Low	High	T/T	C/C	T/T	T/T	G/G
Presence	Low	High	T/T	C/C	T/T	T/T	G/G
Presence	Low	High	T/T	C/C	T/T	T/T	G/G
Presence	Low	High	T/T	C/C	T/T	T/T	G/G
Presence	Low	High	T/T	C/C	T/T	T/T	G/G
Presence	Low	High	T/T	C/C	T/T	T/T	G/G
Presence	Low	High	T/T	C/C	T/T	T/T	G/G
Presence	Low	High	T/T	C/C	T/T	T/T	G/G
Presence	Low	Low	T/T	A/A	G/G	C/C	C/C
Presence	Low	Low	T/C	A/A	G/G	C/C	C/C
Presence	Low	Low	T/C	C/A	T/G	T/C	G/C
Presence	Low	Low	T/C	C/A	T/G	T/C	G/C
Presence	Low	Low	T/C	C/A	T/G	T/C	G/C
Presence	High	High	C/C	C/A	T/G	T/C	G/C
Presence	High	High	C/C	C/C	T/T	T/T	G/G
Presence	High	Low	T/C	A/A	G/G	C/C	C/C
Presence	High	Low	T/C	C/A	T/G	T/C	G/C
Presence	High	Low	T/C	C/A	T/G	T/C	G/C
Presence	High	Low	T/C	C/A	T/G	T/C	G/C
Presence	High	Low	T/C	C/A	T/G	T/C	G/C
Presence	High	Low	T/C	C/A	T/G	T/C	G/C
Presence	High	Low	T/C	C/A	T/G	T/C	G/C
Presence	High	Low	T/C	C/A	T/G	T/C	G/C
Presence	High	Low	C/C	C/A	T/G	T/C	G/C
Presence	High	Low	C/C	C/A	T/G	T/C	G/C

Abbreviations: CIVD, cold-induced vasodilation; NO, nitric oxide; SNP, single-nucleotide polymorphism; Neuro, neurogenic activity; eNO-indep, endothelial NO-independent activity.
Yellow and blue cells represent homozygotes of major and minor alleles, respectively, at each SNP. Light blue cells represent heterozygotes.

Table S8. Relationships among the genotypes of five candidate SNPs defined in the additive model and network of three CIVD-related factors.

			Significant SNPs in the additive model				
CIVD response	Neuro	eNO-indep	rs17113836	rs73619297	rs73619299	rs7321461	rs3832899
Absence	Low	High	A/T	G/A	C/T	A/C	T/-
Absence	Low	High	A/T	G/A	C/T	A/C	T/-
Absence	Low	High	A/T	G/A	C/T	A/C	T/-
Absence	Low	High	A/T	G/A	C/T	A/C	T/-
Absence	Low	High	A/T	G/A	C/T	A/C	T/-
Absence	Low	Low	NA	A/A	T/T	C/C	-/-
Absence	High	Low	A/A	A/A	T/T	C/C	-/-
Absence	High	Low	A/A	G/G	C/C	A/A	T/T
Absence	High	Low	A/A	G/G	C/C	A/A	T/T
Absence	High	Low	A/A	G/G	C/C	A/A	T/T
Presence	Low	High	A/A	A/A	T/T	C/C	-/-
Presence	Low	High	A/A	A/A	T/T	C/C	-/-
Presence	Low	High	A/A	A/A	T/T	C/C	-/-
Presence	Low	High	A/A	A/A	T/T	C/C	-/-
Presence	Low	High	A/A	A/A	T/T	C/C	-/-
Presence	Low	High	A/A	A/A	T/T	C/C	-/-
Presence	Low	High	A/A	A/A	T/T	C/C	-/-
Presence	Low	High	A/A	G/A	C/T	A/C	T/-
Presence	Low	High	A/A	G/A	C/T	A/C	T/-
Presence	Low	High	A/A	G/A	C/T	A/C	T/-
Presence	Low	High	A/A	G/A	C/T	A/C	T/-
Presence	Low	High	A/A	G/A	C/T	A/C	T/-
Presence	Low	High	A/A	G/A	C/T	A/C	T/-
Presence	Low	High	A/A	G/A	C/T	A/C	T/-
Presence	Low	High	A/A	G/A	C/T	A/C	T/-
Presence	Low	High	A/A	G/A	C/T	A/C	T/-
Presence	Low	High	A/A	G/A	C/T	A/C	T/-
Presence	Low	High	A/A	G/A	C/T	A/C	T/-
Presence	Low	High	A/A	G/A	C/T	A/C	T/-
Presence	Low	High	A/A	G/G	C/C	A/A	T/T
Presence	Low	High	A/A	G/G	C/C	A/A	T/T
Presence	Low	High	A/A	G/G	C/C	A/A	T/T
Presence	Low	High	A/A	G/G	C/C	A/A	T/T
Presence	Low	High	A/A	G/G	C/C	A/A	T/T
Presence	Low	High	A/A	G/G	C/C	A/A	T/T
Presence	Low	High	A/A	G/G	C/C	A/A	T/T
Presence	Low	High	A/A	G/G	C/C	A/A	T/T
Presence	Low	High	A/A	G/G	C/C	A/A	T/T
Presence	Low	High	A/A	G/G	C/C	A/A	T/T
Presence	Low	High	A/A	G/G	C/C	A/A	T/T
Presence	Low	High	A/A	G/G	C/C	A/A	T/T
Presence	Low	High	A/A	G/G	C/C	A/A	T/T
Presence	Low	High	A/A	G/G	C/C	A/A	T/T
Presence	Low	High	A/A	G/G	C/C	A/A	T/T
Presence	Low	High	A/A	G/G	C/C	A/A	T/T
Presence	Low	High	A/A	G/G	C/C	A/A	T/T
Presence	Low	High	A/A	G/G	C/C	A/A	T/T
Presence	Low	High	A/A	G/G	C/C	A/A	T/T
Presence	Low	High	A/T	A/A	T/T	C/C	-/-
Presence	Low	High	A/T	A/A	T/T	C/C	-/-
Presence	Low	High	A/T	A/A	T/T	C/C	-/-
Presence	Low	High	A/T	A/A	T/T	C/C	-/-
Presence	Low	High	A/T	A/A	T/T	C/C	-/-
Presence	Low	High	A/T	G/A	C/T	A/C	T/-

Presence	Low	High	A/T	G/A	C/T	A/C	T/-
Presence	Low	High	A/T	G/A	C/T	A/C	T/-
Presence	Low	High	A/T	G/A	C/T	A/C	T/-
Presence	Low	High	A/T	G/A	C/T	A/C	T/-
Presence	Low	High	A/T	G/A	C/T	A/C	T/-
Presence	Low	High	A/T	G/A	C/T	A/C	T/-
Presence	Low	High	A/T	G/A	C/T	A/C	T/-
Presence	Low	High	A/T	G/A	C/T	A/C	T/-
Presence	Low	High	A/T	G/A	C/T	A/C	T/-
Presence	Low	High	A/T	G/A	C/T	A/C	T/-
Presence	Low	High	A/T	G/G	C/C	A/A	T/T
Presence	Low	High	A/T	G/G	C/C	A/A	T/T
Presence	Low	High	A/T	G/G	C/C	A/A	T/T
Presence	Low	High	A/T	G/G	C/C	A/A	T/T
Presence	Low	High	A/T	G/G	C/C	A/A	T/T
Presence	Low	High	A/T	G/G	C/C	A/A	T/T
Presence	Low	High	A/T	G/G	C/C	A/A	T/T
Presence	Low	High	A/T	G/G	C/C	A/A	T/T
Presence	Low	High	T/T	G/A	C/T	A/C	T/-
Presence	Low	High	T/T	G/A	C/T	A/C	T/-
Presence	Low	High	T/T	G/A	C/T	A/C	T/-
Presence	Low	High	T/T	G/G	C/C	A/A	T/T
Presence	Low	High	T/T	G/G	C/C	A/A	T/T
Presence	Low	Low	A/A	G/G	C/C	A/A	T/T
Presence	Low	Low	A/T	G/G	C/C	A/A	T/T
Presence	Low	Low	A/T	G/G	C/C	A/A	T/T
Presence	Low	Low	A/T	G/G	C/C	A/A	T/T
Presence	Low	Low	A/T	A/A	T/T	C/C	-/-
Presence	High	High	A/T	G/A	C/T	A/C	T/-
Presence	High	High	A/T	G/A	C/T	A/C	T/-
Presence	High	Low	A/A	G/A	C/T	A/C	T/-
Presence	High	Low	A/A	G/A	C/T	A/C	T/-
Presence	High	Low	A/A	G/A	C/T	A/C	T/-
Presence	High	Low	A/T	G/A	C/T	A/C	T/-
Presence	High	Low	A/T	G/A	C/T	A/C	T/-
Presence	High	Low	A/T	G/A	C/T	A/C	T/-
Presence	High	Low	A/T	G/A	C/T	A/C	T/-
Presence	High	Low	A/T	G/A	C/T	A/C	T/-
Presence	High	Low	A/T	G/A	C/T	A/C	T/-
Presence	High	Low	A/T	G/G	C/C	A/A	T/T

Abbreviations: CIVD, cold-induced vasodilation; NO, nitric oxide; SNP, single-nucleotide polymorphism; Neuro, neurogenic activity; eNO-indep, endothelial NO-independent activity.
Hyphens indicate indels. Yellow and blue cells represent homozygotes of major and minor alleles, respectively, at each SNP. Light blue cells represent heterozygotes.

Table S9. Correlation estimates of four candidate SNPs with their adjacent SNPs using LDproxy.

RefSNP ID	Position	Alleles	MAF	Distance	*D'*	r^2
rs931740†	5:35222040	T/C	0.433	0		
rs6878684	5:35223842	C/T	0.447	1802	1.000	0.943
rs34232360	5:35224829	A/-	0.447	2789	1.000	0.943
rs17113836†	5:152344712	A/T	0.250	0		
rs80110888	5:152348048	A/G	0.260	3336	1.000	0.951
rs73619297†	13:110967247	G/A	0.409	0		
rs73619299	13:110967456	C/T	0.409	209	1.000	1.000
rs73619293	13:110966788	T/C	0.409	−459	1.000	1.000
rs3832899	13:110968317	T/-	0.409	1070	1.000	1.000
rs7321461	13:110967990	A/C	0.414	743	1.000	0.980
rs112270785	13:110970673	-/G	0.409	3426	0.960	0.922
rs115243474	13:110970675	T/A	0.409	3428	0.960	0.922
rs10144241†	14:65638439	C/A	0.404	0		
rs10133408	14:65638443	T/G	0.404	4	1.000	1.000
rs10133278	14:65638277	T/C	0.404	−162	1.000	1.000
rs11850650	14:65639582	C/T	0.399	1143	1.000	0.980
rs11158583	14:65641686	C/G	0.399	3247	1.000	0.980
rs12889637	14:65639256	C/T	0.385	817	1.000	0.923
rs8012038	14:65640957	G/T	0.385	2518	1.000	0.923
rs35299636	14:65634376	C/T	0.385	−4063	1.000	0.923
rs11158582	14:65634324	C/T	0.385	−4115	1.000	0.923
rs60933199	14:65634016	T/-	0.385	−4423	1.000	0.923
rs12147743	14:65644204	C/T	0.380	5765	1.000	0.904
rs56134334	14:65645107	G/A	0.380	6668	1.000	0.904
rs61989767	14:65645115	T/G	0.380	6676	1.000	0.904
rs56115093	14:65645129	T/C	0.380	6690	1.000	0.904
rs7158173	14:65645250	T/C	0.380	6811	1.000	0.904
rs7158687	14:65645468	T/A	0.380	7029	1.000	0.904
rs8011108	14:65647839	C/T	0.380	9400	1.000	0.904
rs8011246	14:65647885	C/T	0.380	9446	1.000	0.904
rs11851179	14:65627669	G/C	0.370	−10770	1.000	0.868
rs10873187	14:65627466	G/A	0.370	−10973	1.000	0.868
rs12437124	14:65624321	T/C	0.370	−14118	1.000	0.868
rs36120512	14:65624180	A/G	0.370	−14259	1.000	0.868
rs61291180	14:65622841	C/T	0.370	−15598	1.000	0.868
rs36066968	14:65650954	G/T	0.375	12515	0.979	0.848

Abbreviations: SNP, single-nucleotide polymorphism; Position, chromosomal position (NCBI build GRCh37); MAF, minor allele frequency.
Only SNPs with $r^2 > 0.8$ are shown.
† Query SNP.

SI References

1. Sera T, Kohno T, Nakashima Y, Uesugi M, Kudo S. Low-frequency oscillations of finger skin blood flow during the initial stage of cold-induced vasodilation at different air temperatures. J Physiol Anthropol. 2020;39:37.
2. Gabriel SB, Schaffner SF, Nguyen H, Moore JM, Roy J, Blumenstiel B, et al. The structure of haplotype blocks in the human genome. Science. 2002;296:2225–9.
